# Synthesis and Properties of Open Fullerenes Encapsulating Ammonia and Methane

**DOI:** 10.1002/cphc.201701212

**Published:** 2018-01-04

**Authors:** Sally Bloodworth, John Gräsvik, Shamim Alom, Karel Kouřil, Stuart J. Elliott, Neil J. Wells, Anthony J. Horsewill, Salvatore Mamone, Mónica Jiménez‐Ruiz, Stéphane Rols, Urmas Nagel, Toomas Rõõm, Malcolm H. Levitt, Richard J. Whitby

**Affiliations:** ^1^ Chemistry University of Southampton Southampton SO17 1BJ UK; ^2^ School of Physics and Astronomy University of Nottingham Nottingham NG7 2RD UK; ^3^ National Institute of Chemical Physics and Biophysics Akadeemia Tee 23 Tallinn 12618 Estonia; ^4^ Institut Laue-Langevin, CS 20156 38042 Grenoble France

**Keywords:** endofullerenes, inelastic neutron scattering spectroscopy, infra-red spectroscopy, NMR spectroscopy, SpinDynamica

## Abstract

We describe the synthesis and characterisation of open fullerene (**1**) and its reduced form (**2**) in which CH_4_ and NH_3_ are encapsulated, respectively. The ^1^H NMR resonance of endohedral NH_3_ is broadened by scalar coupling to the quadrupolar ^14^ n nucleus, which relaxes rapidly. This broadening is absent for small satellite peaks, which are attributed to natural abundance ^15^N. The influence of the scalar relaxation mechanism on the linewidth of the ^1^H ammonia resonance is probed by variable temperature NMR. A rotational correlation time of *τ_c_*=1.5 ps. is determined for endohedral NH_3_, and of *τ_c_*=57±5 ps. for the open fullerene, indicating free rotation of the encapsulated molecule. IR spectroscopy of NH_3_@**2** at 5 K identifies three vibrations of NH_3_ (*ν*_1_, *ν*_3_ and *ν*_4_) redshifted in comparison with free NH_3_, and temperature dependence of the IR peak intensity indicates the presence of a large number of excited translational/ rotational states. Variable temperature ^1^H NMR spectra indicate that endohedral CH_4_ is also able to rotate freely at 223 K, on the NMR timescale. Inelastic neutron scattering (INS) spectra of CH_4_@**1** show both rotational and translational modes of CH_4_. Energy of the first excited rotational state (*J=*1) of CH_4_@**1** is significantly lower than that of free CH_4_.

## 1 Introduction

The potential for encapsulation of an atom or small molecule within the cavity of spherical fullerenes, has long been recognized.^1^ The inert and highly symmetric, three‐dimensional environment of the cavity, means that enclosed (endohedral) species are expected to behave much as they would in the very low pressure gas state, with preservation of free rotation down to cryogenic temperatures.^2^ Although direct synthesis of endohedral metallofullerenes,^3^ and fullerenes containing individual atoms (noble gas@C_60_ 4 and the remarkable N@C_60_ 5) is possible in very low yield, currently the only high‐yielding route to small molecule endofullerenes is via the process of “molecular surgery”^6^ whereby chemical transformations are used to open a hole in the fullerene, a molecule is inserted, and a further series of reactions is then used to suture the opening and reform the pristine fullerene shell. To date, this has only been achieved for the incorporation of H_2_,^7^ H_2_O^8^ and HF,^9^ as well as their related isotopologues.

Encapsulated H_2_ and H_2_O have proven particularly interesting due to the interaction of their nuclear spin and rotational states. Due to the Pauli principle, the *ortho*‐ (nuclear spins aligned) and *para*‐ (nuclear spins opposed) allotropes are limited to odd and even rotational states, respectively. For H_2_O@C_60_, isolation contributes to long lived *ortho*‐ and *para*‐spin states and has allowed their interconversion and physical phenomenon such as spin dependent electric polarizability to be measured.^10^

A range of larger molecules including N_2_,^11^, ^12^, ^13^ O_2_,^14^ CO,^13^, ^15^ NH_3_,^16^ CO_2_,^11^ CH_4_,^17^ CH_2_O,^18^, ^19^ CH_3_OH,^18^ HCN,^19^, ^20^ and HCCH^20^ have been incorporated into open fullerenes via a larger opening in the shell, but closure to reform the pristine fullerene cage has not yet been achieved for these examples. Although the high symmetry of the closed cage is lost in these open derivatives, many other properties including isolation are retained.^21^

Encapsulated NH_3_ and CH_4_ are of particular interest because (as with H_2_O and H_2_) their symmetry leads to interaction between the rotational (*J*) and nuclear spin (*I*) quantum states. Methane exists as three nuclear spin isomers (with overall spin 2, 1 and 0) such that the ground rotational state (*J=*0) can only be occupied by *meta*‐CH_4_ (*I*=2), whereas the *J=*1 state is limited to *ortho*‐CH_4_ (*I*=1). Ammonia exists as the *para* (*I*=^1^/_2_) and *ortho* (*I*=^3^/_2_) nuclear spin isomers which dictate permitted transitions between rotational and vibrational states, with the “umbrella” inversion being particularly interesting.

**Figure 1:**
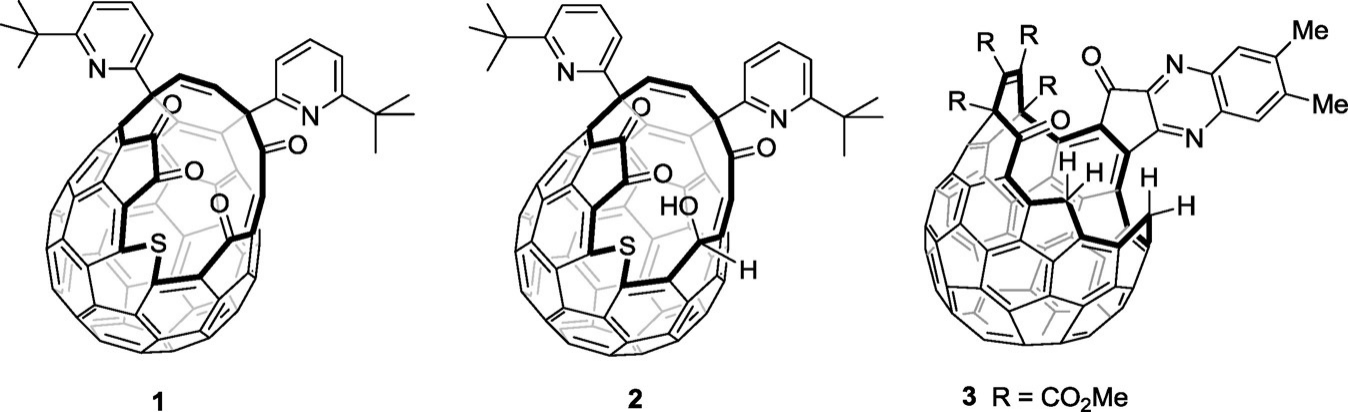
Open fullerenes.

Herein we describe encapsulation of CH_4_ in open fullerene **1**, and NH_3_ in open fullerene **2** (Figure 1), and some properties of the resulting species CH_4_@**1** and NH_3_@**2**. Encapsulation of H_2_O,^12^ CH_2_O,^18^ CH_3_OH,^18^ N_2_,^11^ and CO_2_ 11 in **1** has been reported, as has encapsulation of CH_2_O,^18^ CO_2_,^11^ O_2_ 14 and N_2_ 11 in **2** following reduction of **1** to avoid loss of the endohedral molecule. Both NH_3_ and CH_4_ have previously been incorporated in the unrelated open fullerene **3**.^16^, ^17^

## 2 Results and Discussion

### 2.1 Synthesis of CH_4_@ Open Fullerene

**Scheme 1:**
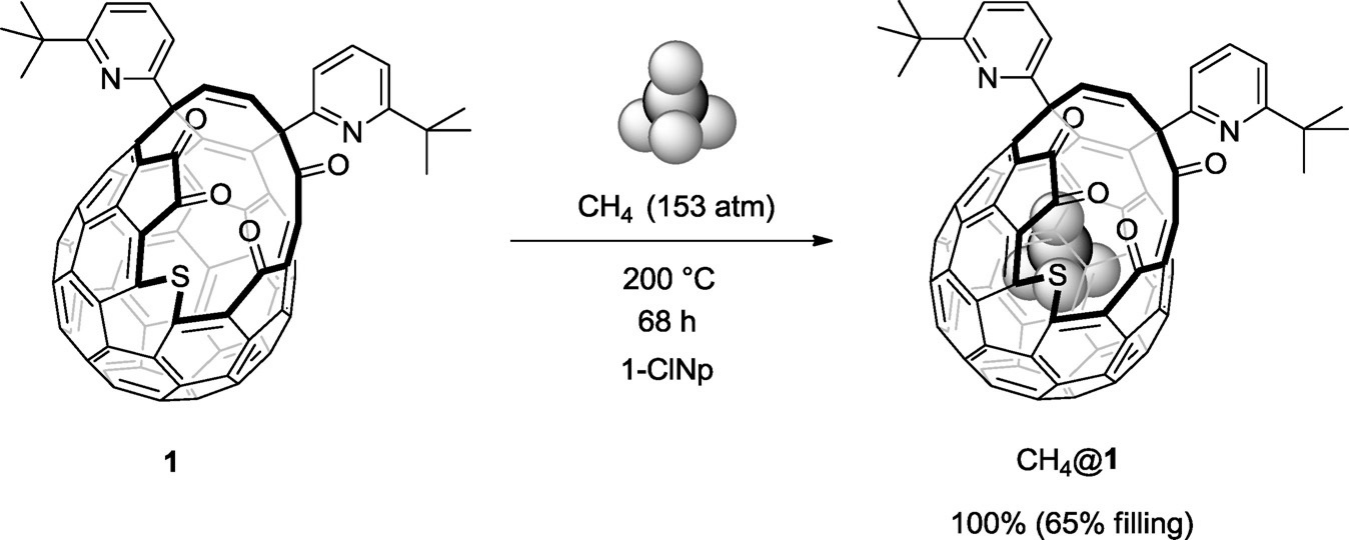
Filling of fullerene **1** with methane.

Heating the open‐cage fullerene **1** 12 at 200 °C for 68 h, under 153 atm of methane gave CH_4_@**1** with 65 % encapsulation of the endohedral molecule in quantitative yield (Scheme 1). The filling factor of CH_4_@**1** was established by comparison of integrated resonances for CH_4_ and an exohedral alkene proton in the ^1^H NMR spectrum. For comparison, CH_4_@**3** was prepared by Iwamatsu and co‐workers in 20 % yield and 39 % incorporation, after 20 h at 200 °C under 190 atm of CH_4_.^17^

**Figure 2:**
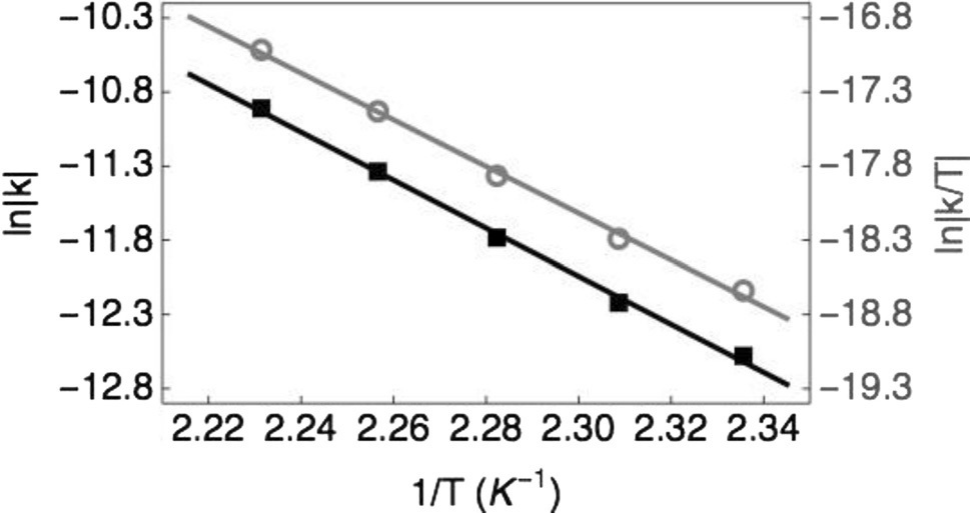
Arrhenius (black squares) and Eyring (grey circles) plots for thermal first‐order dissociation of CH_4_@**1**.

The rate of loss of CH_4_ from CH_4_@**1** was measured at several temperatures between 428 and 448 K and displayed the expected 1^st^ order kinetics. From the linear Arrhenius and Eyring plots (Figure 2), an activation energy of 134.6±5.0 kJ mol^−1^ and pre‐exponential factor log(A) of 10.9 was determined. The latter is low for a unimolecular reaction, but comparable for those previously observed for loss of endohedral atoms and molecules from endohedral fullerenes.^13^ The enthalpy and entropy of activation for CH_4_ loss were determined to be Δ*H* ^≠^=131.0±5.0 kJ mol^−1^ and Δ*S* ^≠^=−47.0±11.2 J K^−1^ mol^−1^ giving a Δ*G* ^≠^ at 165 °C of 151.5±0.1 kJ mol^−1^. The values are in good agreement with those calculated by Density Functional Theory methods (Δ*H* ^≠^=135.9 kJ mol^−1^; Δ*S* ^≠^=−31.5 J K^−1^ mol^−1^; Δ*G* ^≠^=149.7 kJ mol^−1^ at 165 °C). The negative entropy of activation is unusual for a dissociative reaction, but reflects the loss of rotational and translational degrees of freedom as the endohedral molecule is constrained by its passage through the orifice.

### 2.2 Physical Properties of CH_4_@ Open Fullerene

**Figure 3:**
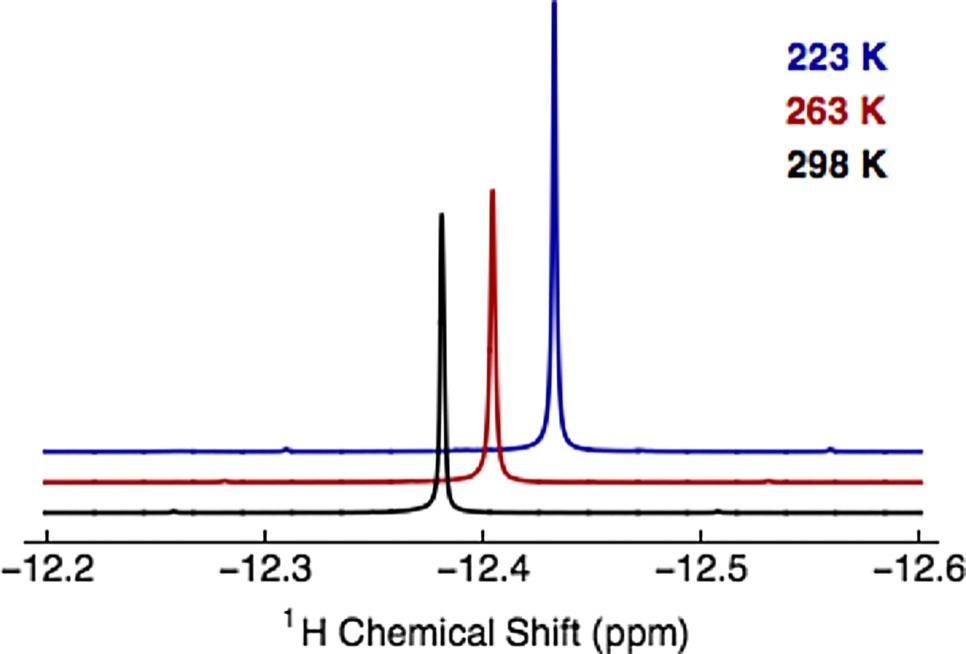
A section of the experimental ^1^H NMR spectrum of 12.0 mm CH_4_@**1** in degassed CDCl_3_ solution acquired at 11.7 T (500 MHz) with 64 transients at each temperature; 223 K, 263 K and 298 K. ^13^C satellites for the endohedral methane resonance are visible in each spectrum.

The ^1^H resonance of endohedral CH_4_ in CH_4_@**1** appears as a sharp singlet at *δ*=−12.59 ppm (500 MHz, 1,2‐dichlorobenzene‐*d*_4_) or −12.33 ppm (500 MHz, CDCl_3_), compared with *δ*=2.17 ppm for gaseous CH_4_.^22^ The ^1^H spectrum was acquired with a pulse delay (d1) of 30 s. following measurement of the experimental ^1^H spin‐lattice relaxation curve for CH_4_@**1** which shows single exponential decay with a time constant of *T*_1_=7.53±0.03 s. (see Supporting Information). Variable temperature proton NMR showed no line broadening down to 223 K (Figure 3) indicating free rotation of the endohedral methane on the NMR timescale. Temperature‐dependence of the ^1^H chemical shift is unexplained, but may be due to one or more configurations of different energy, in fast exchange on the NMR timescale.

The ^13^C resonance of endohedral CH_4_ appears as a pentet, centred at *δ*=−19.47 ppm with ^1^ *J*_CH_=124.8 Hz, in the proton‐coupled ^13^C NMR spectrum (125.7 MHz, 1,2‐dichlorobenzene‐*d*_4_) compared with *δ*=−8.65 ppm^22^ and ^1^ *J*_CH_=125.3 Hz^23^ for gaseous CH_4_.

Although CH_4_@**1** is stable to loss of CH_4_ at room temperature, the “empty” open‐cage fullerene component **1** (i.e. 35 % of the material) was found to readily encapsulate water upon exposure to the atmosphere. Rapid exchange of a water molecule between the *endo*‐ and exohedral environments has been reported by Murata and co‐workers^12^ and is characterized by a very broad ^1^H resonance which we detect at *δ*=−11.62 ppm in 1,2‐dichlorobenzene‐*d*_4_. Dry samples of the inseparable 65:35 mixture of CH_4_@**1:1** were most readily obtained by removal of water as its azeotrope with THF.

**Figure 4:**
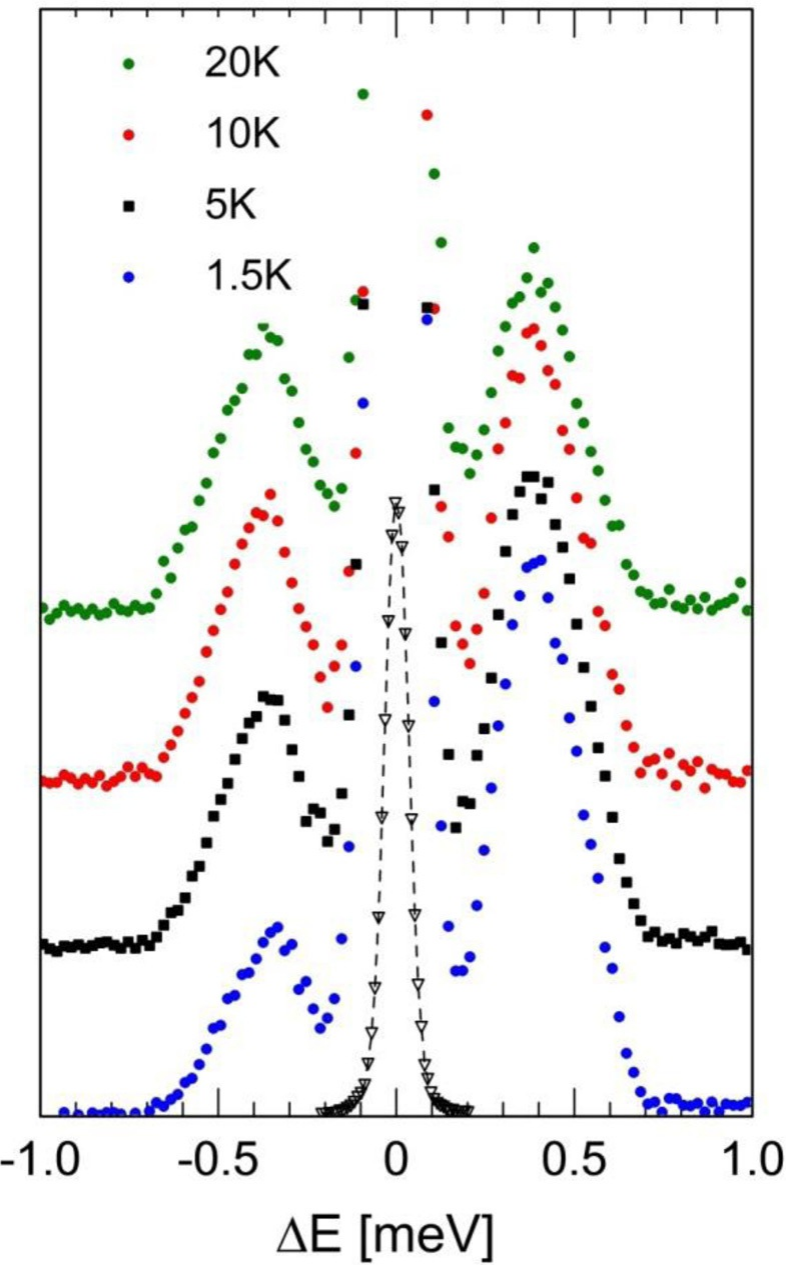
The INS spectrum of CH_4_@**1** recorded on the time of flight (t.o.f.) spectrometer IN6, *T=*1.5, 5, 10, 20 K. Data with open black triangles depicts the elastic line with an intensity scaling factor ×0.04.

**Figure 5:**
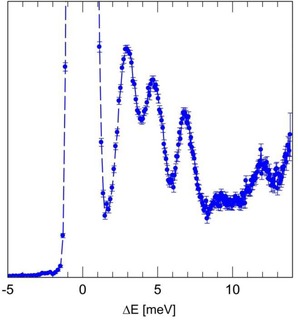
The INS spectrum of CH_4_@**1** recorded on the t.o.f. spectrometer IN4c. λ_n_=2.3 Å and *T=*1.6 K.

**Figure 6:**
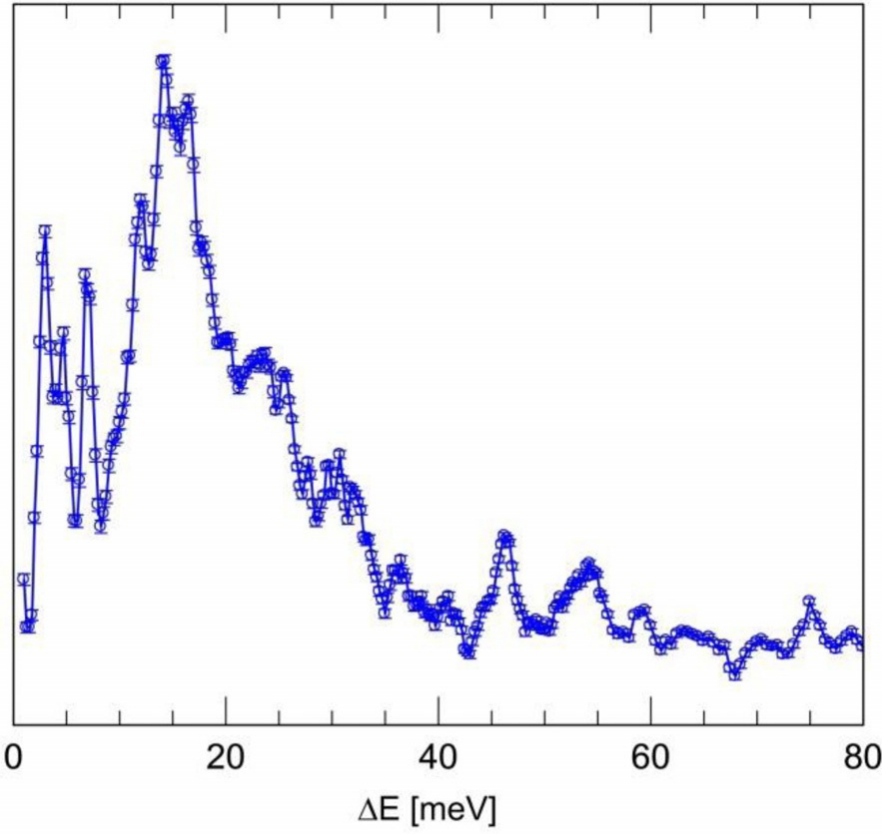
The INS spectrum of CH_4_@**1** recorded on IN1‐Lagrange. *T=*2.7 K.

INS spectra for dry CH_4_@**1,** covering a wide range of energy transfer, were recorded using the IN‐1 Lagrange, IN4c and IN6 spectrometers (Figures 4–6). INS spectra from a “blank” sample of open fullerene **1** were subtracted from those recorded on the sample containing CH_4_.

IN6 spectra (Figure 4) are presented, recorded with temperatures 1.5, 5, 10 and 20 K. A matching pair of inelastic peaks centred on |Δ*E*|=0.4 meV are observed in NE gain and NE loss. With FWHM (full‐width‐half‐maximum) linewidths of approximately 0.27 meV, the inelastic features are substantially broader than the resolution linewidth (FWHM 0.08 meV, as measured by the elastic line at zero energy transfer).

The IN4c spectrum (Figure 5) was recorded with incident wavelength λ_n_=2.3 Å and *T=*1.6 K. A trio of peaks is observed in NE loss with energy transfer, Δ*E*=2.95, 4.65 and 6.85 meV. Finally, the IN1‐Lagrange spectrum (Figure 6) provides access to a wide range of energy, *T=*2.7 K. The same trio of low energy peaks observed on IN4c is evident, as well as a broad band at higher energy loss (Δ*E*=≈15 meV). Narrower components are evident within this broad band and indeed at higher energies.

The CH_4_ molecule is confined within the small space defined by the fullerene cage, so in addition to rotational degrees of freedom, the molecule also exhibits quantised translation. Analogously to INS investigations on other small molecule endofullerenes such as H_2_@C_60_ and H_2_O@C_60_,^24^ it is expected that the INS spectra of CH_4_@**1** will comprise peaks arising from transitions among the quantised rotational and translational eigenstates of CH_4_ confined in its cage. However, unlike H_2_@C_60_ and H_2_O@C_60_, the open‐cage environment of CH_4_@**1** means the cage potential experienced by the CH_4_ rotor lacks symmetry and is anisotropic.^25^

As a quantum rotor possessing four indistinguishable ^1^H nuclei (fermions), the permissible eigenstates of CH_4_ are classified by the Pauli Principle and we identify nuclear spin isomers for which rotational and nuclear spin eigenstates are entangled. In the free‐rotor limit (as applies to molecule of CH_4_ in the gas phase), the ground rotational state with rotational quantum number *J=*0 has A_1_ symmetry and total nuclear spin *I*=2. The first rotational excited *J=*1 state has F_1_ symmetry and *I*=1, with energy 1.29 meV. The second excited state with *J=*2 has energy 3.89 meV and comprises a degenerate pair of states, one with F_2_ symmetry and *I*=1, the second with E symmetry and *I*=0. The *J=*3 rotational state has energy 7.80 meV and comprises a trio of degenerate states with *I*=1 and *I*=2.^26^ While INS peaks of CH_4_@**1** are observed in the complementary energy range below 10 meV (Figures 4–6), it is evident that they do not conform to those of a free‐rotor. Notably, the 0.4 meV excitation observed on IN6 (Figure 4) is much lower than the *J=*1 level of the free‐rotor. Indeed by analogy with investigations of hindered CH_4_ rotors,^26^, ^27^ where the energy of the *J=*1 state is significantly reduced from its free‐rotor value, we may assign the pair of |Δ*E*|=0.4 meV peaks to the *J=*0 to 1 rotational transition. The peaks at Δ*E*=2.95, 4.65 and 6.85 meV (Figures 5 and 6) are consistent with examples in the literature of *J=*0 to 2,3 transitions for hindered CH_4_.^26^ However, a full assignment of the higher energy rotational peaks is beyond the scope of this preliminary report and will be the subject of a future publication. Nevertheless, the pattern of low energy peaks appears to be similar to that found for CH_4_ in other hindered environments.^26^, ^27^

The ground to first excited state translational excitations of H_2_, H_2_O and HF confined in C_60_ are observed in the range 9≤Δ*E*≤25 meV.9a, 24d, 28 The precise values depend sensitively on the rotor mass and the cage potential. Additionally, given the latter is anisotropic for this open endofullerene, the degeneracy of the translational states is lifted and we expect to observe three translational peaks with different energy in the INS spectrum. We tentatively assign the broad band centred on 15 meV to these non‐degenerate translations and superimposed are some of the higher energy rotational states.

The anisotropy of the cage potential lifts the rotational degeneracy,^25^ leading to a splitting of the respective *mJ* sub‐states. We find evidence for this in the IN6 spectrum (Figure 4), where the inelastic peaks are significantly broader than the resolution function, indicating unresolved fine structure. That these peaks are inhomogenous and possess multiple components is indicated by the significant asymmetry of the NE peak at the lowest temperature 1.5 K, compared with the more symmetric shape observed at higher temperature. As determined by Boltzmann statistics, at 1.5 K different components on the high and low energy side of the 0.4 meV NE gain peak will differ in amplitude by a factor of order 3. This theoretical factor is consistent with the observed NE gain peak shape. At the higher temperatures these unresolved components have more equilibrated Boltzmann factors, as observed. Therefore, we tentatively assign the excess width of the *J=*0 to 1 rotational peaks to rotational fine structure.

On the timescale of the INS experiments (hours) we did not notice any significant changes which might be attributable to a change in the population of the *J=*0 (*m*‐CH_4_) and *J=*1 (*o*‐CH_4_) states, following thermal equilibration. It has been reported that conversion between these states for CH_4_ in an argon matrix, or in the interstices of C_60_ has *t* ^1^/_2_≈1.5–2.5 h.^26^, ^29^

### 2.3 Synthesis of NH_3_@ Open Fullerene

DFT calculations gave the activation free energy for entry of ammonia into open fullerene **1** at STP as 62.3 kJ mol^−1^, higher than that for water (30.7 kJ mol^−1^) but indicating that both will enter rapidly at room temperature nonetheless. The free energy of binding of ammonia in **1** was calculated to be 25 kJ mol^−1^ more favorable than that for water. We were pleased to find that exposure of a sample of fullerene **1** in CDCl_3_ to a 16 % aqueous ammonia solution led to an 85:15 molar ratio of NH_3_@**1** and H_2_O@**1** by ^1^H NMR, the spectrum displaying broad peaks at −12.44 and −11.52 ppm, respectively, indicating selective encapsulation of ammonia in accord with the calculation of binding energies. In order to avoid contamination with H_2_O‐containing species, we switched to a methanolic solution of ammonia as methanol is too large to enter **1** at room temperature.^18^ To our delight, rapid formation of NH_3_@**1** was observed by NMR although attempts to isolate NH_3_@**1** gave only the empty open fullerene (**1**). In contrast, isolation of NH_3_@**3** (the only previously reported NH_3_@open fullerene species) was achieved by column chromatography with ammonia loss occurring only after several months at −10 °C.^16^ Our observation of the instability of NH_3_@**1** to rapid ammonia loss at room temperature is in accord with Murata's conclusion^11^ that **1** behaves as if it has a larger orifice than **3**, despite the cage‐opening being of nominally the same size, that is, a 17‐member ring.

The selective reduction of a carbonyl group on the orifice of **1** to afford **2** has been used to block the escape of O_2_, N_2_ and CO_2_ guests from the fullerene cage.^11^, ^14^ We therefore sought to develop conditions for the encapsulation of ammonia by host fullerene **1**, with subsequent trapping of the endohedral NH_3_ by in situ reduction of a rim carbonyl group to afford NH_3_@**2**.

**Scheme 2:**
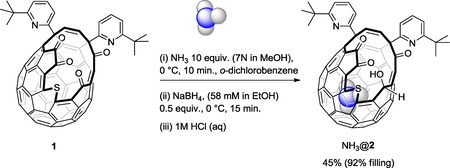
Synthesis of NH_3_@**2**.

Treatment of a solution of **1** in 1,2‐dichlorobenzene with 10 equiv of a 7 n solution of NH_3_ in methanol at 0 °C, followed by reduction with NaBH_4_, afforded NH_3_@**2** in 45 % yield with 92 % encapsulation of NH_3_ (Scheme 2). The filling factor was calculated by comparison of the integrated ^1^H resonance of the endohedral molecule, with that of an exohedral alkene proton. We found that the selective reduction step worked better in the 1,2‐dichlorobenzene solvent reported by Murata,^11^ than in chloroform. The ammonia encapsulation step was found to be sensitive to the period of exposure to NH_3_, with an optimal reaction time of 10 min. Lower NH_3_ encapsulation results from a shorter period (5 min. or 1 min. exposure to NH_3_ gives 80 % or 30 % filling, respectively), but with a reaction time >10 min. we observed the formation of multiple fullerene derivatives by ^1^H NMR. It is probable that these are hemiaminal by‐products since treatment with 1 m HCl (aq.) returns the mixture cleanly to a single compound whose ^1^H NMR spectrum matches that of **1**. The formation of hemiaminal products in competition with NH_3_ encapsulation is likely to limit the filling by “blocking” the fullerene orifice but, importantly, the use of 1 m HCl (aq.) to quench the two‐step (encapsulation/reduction) procedure allows a clean mixture of NH_3_@**2** and starting material **1** to be obtained. Unsurprisingly the encapsulation/reduction was found to be somewhat capricious, giving filling factors for NH_3_@**2** in the range 74–92 % under nominally identical reaction conditions. Pure NH_3_@**2** (74–92 % filled) was readily obtained by column chromatography.

### 2.4 Physical Properties of NH_3_@ Open Fullerene

**Figure 7:**
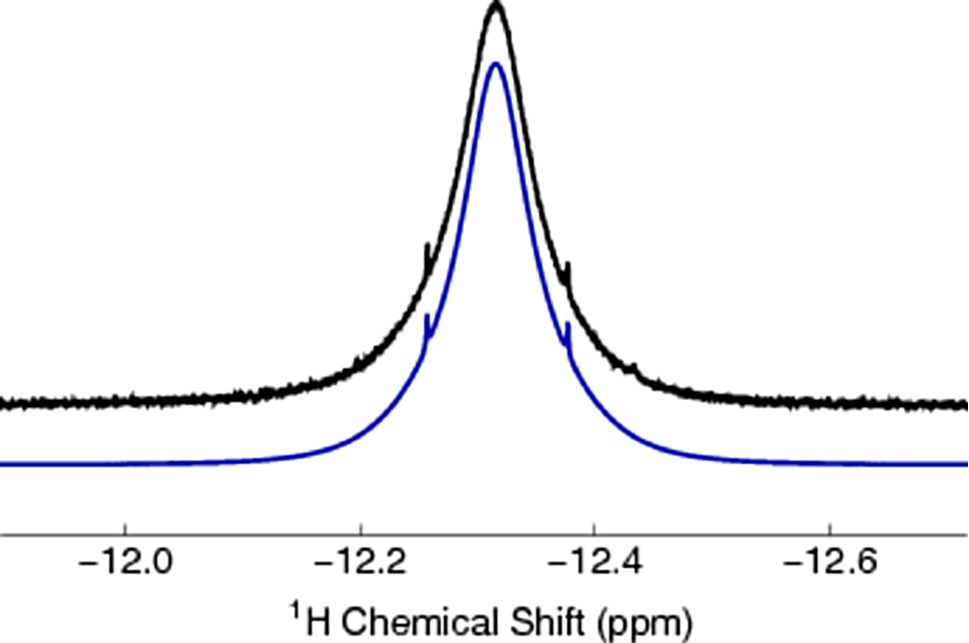
Black line: A section of the experimental ^1^H NMR spectrum of 17.6 mm ^14^NH_3_@**2** in degassed [D_2_]dichloromethane solution acquired at 11.7 T (500 MHz) and 25 °C with 64 transients. Blue line: simulated spectrum obtained using the following parameters: |J_14NH_|=42.6 Hz, *T*_1_(^14^N)=2.66 ms.

The NH_3_ proton resonance of NH_3_@**2** was observed experimentally as a broad peak at *δ*=−12.35 ppm (500 MHz, [D_2_]dichloromethane) with a 38.43 Hz linewidth (Figure 7). The spectral wings are attributed to natural abundance ^15^NH_3_@**2** (approx. 0.3 % intensity), shifted in frequency by a secondary isotope effect (1.5 ppb) and separated by |*J*_15NH_|=59.8 Hz, as was confirmed by independent synthesis of ^15^NH_3_@**2** according to the method described above.

The broad proton peak of ^14^NH_3_, and the very narrow proton peak of the ^15^NH_3_ isotopologue, provides compelling evidence that the line broadening in the ^14^NH_3_ case is due to rapid quadrupolar relaxation of the ^14^N coupling partner. This broadens the ^14^NH_3_ proton resonance through the scalar relaxation of the second kind (SR2K) mechanism.^30^ This broadening mechanism is negligible for the ^15^N isotopologue, since the ^15^N spin‐lattice relaxation is on a much longer timescale, due to the absence of an efficient quadrupolar relaxation mechanism for ^15^N.

The lineshapes are well simulated using *SpinDynamica*,^31^, ^32^ using a ^1^H‐^14^ n scalar coupling of |*J*_14NH_|=42.6 Hz and ^14^N quadrupolar relaxation with a spin‐lattice relaxation time *T*_1_(^14^N)=2.66 ms (Figure 7).

The ^14^N quadrupolar relaxation may be treated by assuming isotropic rotational diffusion. We define the quadrupolar coupling constant by [Eq. (1)]:

(1) $$\omega_{Q}=\frac{e^{2}qQ}{2I(2I-1)\hbar }$$

where *I*=1 for ^14^N, e*Q* is the electric quadrupolar moment of the nitrogen nucleus, and e*q* is the electrical field gradient at the deuterium nucleus.^33^ The Frobenius norm of the quadrupole coupling tensor may be written as [Eq. (2)]:

(2) $$\left|\right|A_{Q}\left|\right|=\omega_{Q}{\left(\frac{1}{2}\left(3+\eta^{2}\right)\right)}^{\frac{1}{2}}$$

where *η* is the biaxality (asymmetry) parameter of the electric field gradient tensor. The spin‐lattice relaxation rate constant for quadrupolar relaxation, assuming isotropic rotational diffusion and the extreme narrowing limit, is given by [Eq. (3)]:^34^

(3) $$T_{1}^{-1}=\frac{1}{5}\left(2I-1\right)\left(2I+3\right)\left|\right|A_{Q}|{|}^{2}\tau_{C}$$

which, for *I*=1, is equal to [Eq. (4)]:

(4) $$T_{1}^{-1}=\left|\right|A_{Q}|{|}^{2}\tau_{C}$$

The nuclear quadrupole coupling constant for ^14^NH_3_ has been estimated by microwave spectroscopy to be: *ω*_Q_/2*π*=2.05 MHz,^35^ with a biaxality parameter of *η*=0.^36^ The ^14^N *T*_1_ value of 2.66 ms, as inferred from the lineshape of the ^1^H NMR spectrum, leads to an estimate of the rotational correlation time for endohedral ammonia molecules of *τ_c_*=1.5 ps. If the overall tumbling of the fullerene cage is at least one order of magnitude slower as expected,^37^ this would indicate that NH_3_ is (essentially) rotating freely. We therefore calculated the rotational correlation time (*τ_c_*) of open fullerene **2** from the experimental relaxation time constant *T*_1_(^13^C)=0.54±0.06 s. of the methine carbon located on the orifice of **2**, using Equation (5) to define relaxation of the ^13^C(H)OH nucleus, as applicable for extreme‐narrowing isotropic rotational tumbling, dominated by the ^13^C‐^1^H dipolar relaxation mechanism:

(5) $$T_{1}^{-1}\left(13C\right)=\frac{3}{2}\omega_{CH}^{2}\tau_{C}$$

where *ω*_CH_ is the dipole‐dipole coupling constant for the interaction between the carbon and proton nuclei, and *τ_c_* is the overall rotational correlation time for **2**.

By assuming an internuclear ^13^C‐^1^H distance of 108.9 pm, which corresponds to a dipole coupling constant of *ω*_CH_/2*π*=−23.4 kHz, we obtain an estimate of *τ_c_*=57±5 ps. for the open fullerene **2**, which is more than an order of magnitude longer than the rotational correlation time for the endohedral ammonia molecule in NH_3_@**2**.

**Figure 8:**
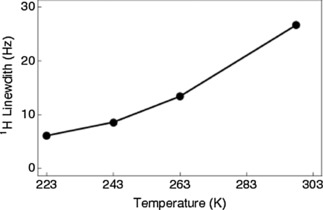
Experimental linewidth for the ^1^H resonance of ^14^NH_3_@**2** plotted as a function of temperature. 64 transients were acquired per data point at a magnetic field of 11.7 T (500 MHz) using an 8 mm sample of ^14^NH_3_@**2** in degassed chloroform‐*d*_3_ solution.

Variable temperature solution proton NMR on NH_3_@**2** showed that the NH_3_ line broadens as the temperature is increased (Figure 8). The increase in linewidth with temperature is consistent with the SR2K mechanism: a temperature increase leads to a shorter rotational correlation time *τ_c_*, which leads to slower quadrupolar relaxation for the ^14^N nucleus, according to Equation (4). This leads in turn to a less effective averaging of the *J*_NH_ splittings, and hence to a broader proton peak.

The experimental ^1^H spin‐lattice relaxation curve for NH_3_@**2** shows single exponential decay with a time constant of *T*_1_=5.05±0.02 s, (see Supporting Information for all spin‐lattice relaxation curves).

**Figure 9:**
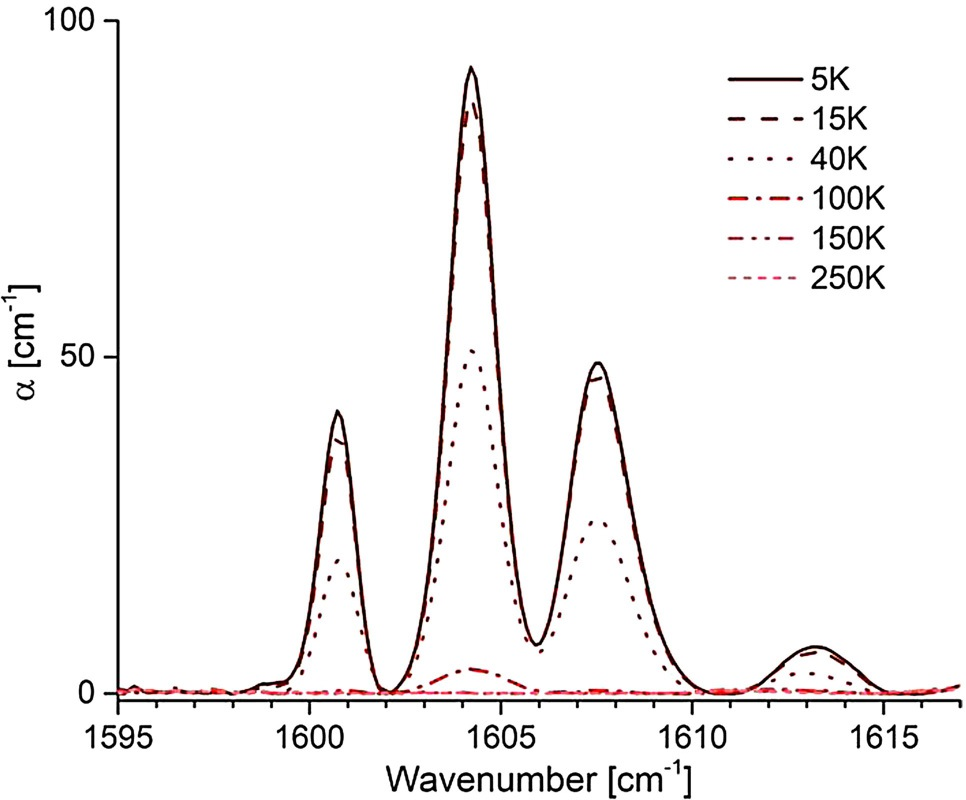
IR spectra of NH_3_@**2** recorded between 5 and 250 K, in the regions around 1600 cm^−1^.

**Figure 10:**
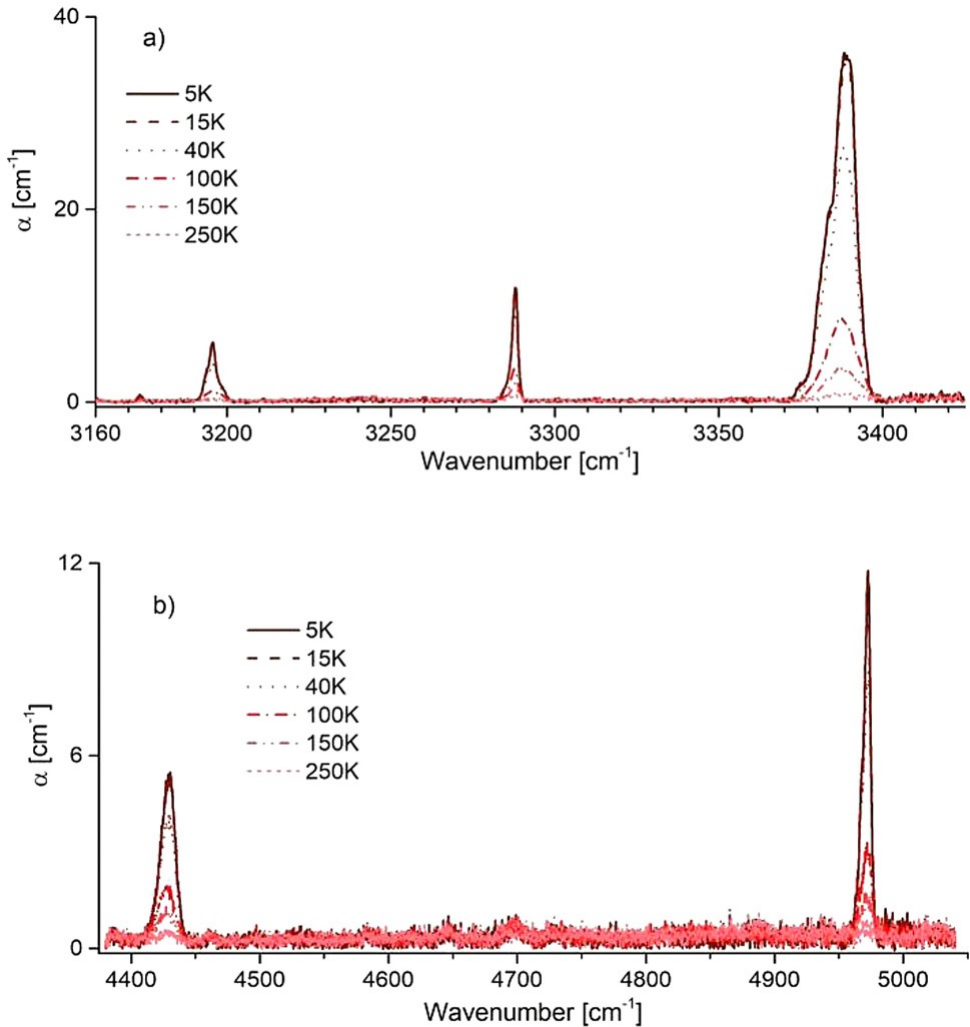
IR spectra of NH_3_@**2** recorded between 5 and 250 K, in the regions around (a) 3300 cm^−1^ and (b) 4700 cm^−1^.

We measured the IR spectra of endofullerene NH_3_@**2** and the empty open‐cage species **2** between 600 and 9000 cm^−1^ in the temperature range 5 K to 300 K. The spectra of NH_3_@**2** show clear peaks present only in NH_3_@**2** and not in **2** in three spectral regions, around 1604 cm^−1^, 3300 cm^−1^, and 4700 cm^−1^ (Figures 9 and 10). The ≈1604 cm^−1^ region contains a cluster of four well‐resolved peaks, each of which may be fitted well with a Gaussian shape (Figure 9). The other two spectral regions contain peaks at 3196, 3288 and 3380 cm^−1^ (Figure 10 a), and 4430 and 4970 cm^−1^ (Figure 10 b). It should be noted that the spectral regions between 900–950, 1550–1950 and 3400–3550 cm^−1^ are obscured by strong fullerene absorption.

**Figure 11:**
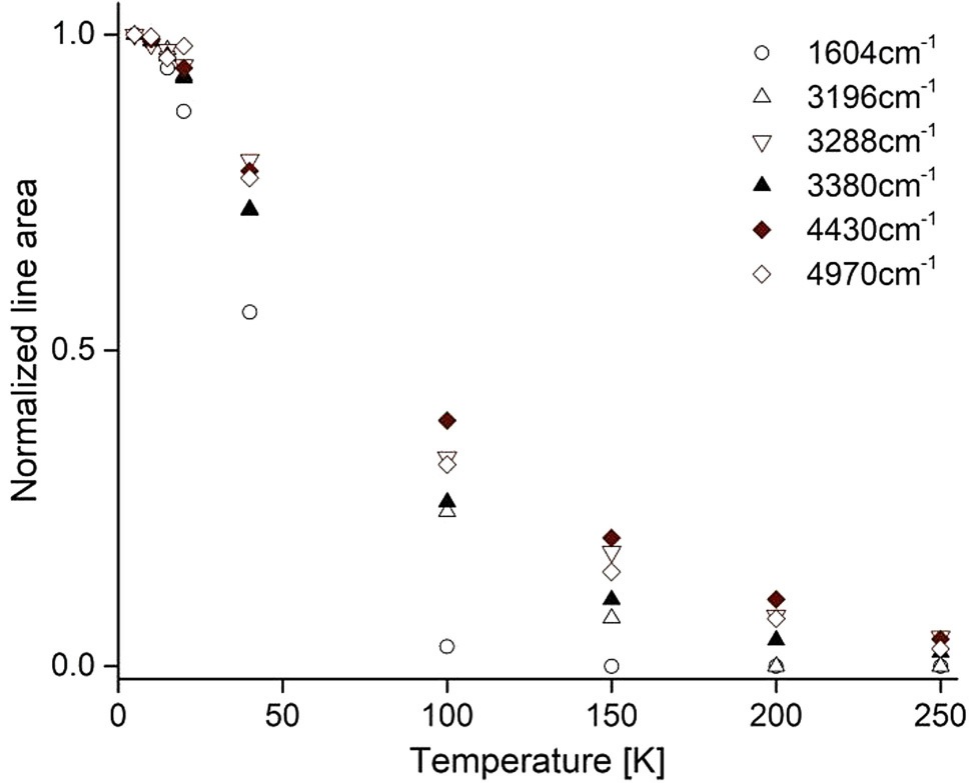
Temperature dependence of NH_3_@**2** normalized IR absorption line areas. Peaks are labeled by their frequencies. The normalized area of the 1604 cm^−1^ peak is the normalized sum of four peak areas from Figure 9.

The intensity of all peaks decreases rapidly as the temperature is increased above ≈10 K (Figure 11), with a particularly strong decrease with temperature observed for the ≈1604 cm^−1^ peak cluster. However, the members of this peak cluster always display the same *relative* intensities.

In the gas phase, NH_3_ has two vibrations with hydrogen atoms moving in the triangular plane (*ν*_2_ and *ν*_4_), and two vibrations with out‐of‐plane motions of hydrogen atoms *ν*_1_ and *ν*_3_ (Table 1).^38^ The *ν*_1_ and *ν*_2_ vibrations are non‐degenerate, while the *ν*_3_ and *ν*_4_ vibrations are doubly degenerate in the gas phase. The following tentative assignments of the NH_3_ absorption peaks in NH_3_@**2** may be made; the cluster of peaks at ≈1604 cm^−1^ is assigned to the *ν*_4_ vibration, redshifted by ≈24 cm^−1^ with respect to the same mode in NH_3_ gas. The 3196 cm^−1^ peak is assigned to the fundamental *ν*_1_ vibration, redshifted by 141 cm^−1^ with respect to the same mode in NH_3_ gas. The 3288 cm^−1^ and 3380 cm^−1^ peaks are assigned to the *ν*_3_ vibration. We postulate that the double degeneracy of the *ν*_3_ mode is lifted by the asymmetric environment of the open cage. The mean frequency of these two peaks is 3334 cm^−1^, indicating a redshift of 109 cm^−1^ with respect to the same mode in NH_3_ gas. The peak at 4430 cm^−1^ is difficult to attribute to a known mode or feasible combination mode of NH_3_ alone, and we tentatively assign it to a combination mode involving coupled vibrations of the endohedral molecule and a mode of the enclosing cage. The high‐frequency peak at 4970 cm^−1^ is tentatively attributed to a combination mode, involving the ≈1604 cm^−1^ *ν*_4_ vibration and the 3380 cm^−1^ component of the *ν*_3_ vibration.

**Table 1:** Normal modes of NH_3_, their irreducible representations in point group *C*3*ν*, frequencies in the gas phase and measured frequencies in NH_3_@**2**.

Mode	Γ*i* (*C*_3_ *ν*)	*ω* gas (cm^−1^)^39^, ^40^	*ω* NH_3_@**2** (cm^−1^)	(*ω* NH_3_@**2**−*ω* gas)/ *ω* gas
*ν*_1_	*A*_1_	3337	3196	−0.042
*ν*_2_	*A*_1_	950	–	–
*ν*_3_	*E*	3443	3288 3380	−0.045 −0.018
*ν*_4_	*E*	1628	1604	−0.015
			–	–

Gaseous NH_3_ has a *ν*_2_ vibrational mode at 950 cm^−1^. No analogous peak is observed in the spectrum of NH_3_@**2**. The absence of this peak is probably due to the strong fullerene absorption in this spectral region.

No obvious fine structure is observed on the IR peaks, with the exception of the ≈1604 cm^−1^ peak cluster. The absence of rotational fine structure indicates that the potential generated by the confining asymmetric cage quenches the rotational freedom of the NH_3_ molecule, at least for the small number of states that are significantly populated at cryogenic temperatures.

The strong decrease in peak intensities with increasing temperature (Figure 11) implies the existence of many levels above the ground state which become thermally populated at the expense of the ground state population. These higher levels presumably have a complex rotational/translational fine structure. As a result, the spectral absorption at elevated temperature is distributed over a very large number of unresolved spectral peaks, so that all identifiable spectral features disappear at high temperature.

An exception to the absence of fine structure is the peak cluster at ≈1604 cm^−1^, which is tentatively assigned to the *ν*_4_ vibration (Figure 9). Since the relative intensities of the sharp components appear to be temperature‐independent, the fine structure must be due to a splitting of the excited state energy levels, with the ground state remaining unsplit. The origin of this splitting is unknown, but might be due to rotational fine structure, or a tunnelling process between two or more potential minima. However, it is unclear why such structure is not clearly displayed for all of the other peaks and, at present, we do not have a definitive explanation for the fine structure around ≈1604 cm^−1^.

## 3 Conclusions

We have prepared and characterized open fullerenes encapsulating ammonia and methane. The encapsulated methane and ammonia display long ^1^H spin‐lattice relaxation times at room temperature, of 7.5 and 5.1 s. respectively. The variable temperature ^1^H NMR spectra indicate that both endohedral molecules rotate freely within the cages at 223 K, on the NMR timescale.

In the INS spectra of CH_4_@**1** we find the first rotational peak at 0.4 meV and translational energy at approximately 15 meV for CH_4_. This is in qualitative agreement with CH_4_ entrapped in interstitial sites of the C_60_ crystal,^26^ where the *J=*0 to 1 rotational transition is centred on 0.6 meV, and the translational peak is centred on 10.9 meV. The differences are indicative of a stronger crystal field potential, and stronger confinement of the methane in CH_4_@**1**. We did not observe properties resulting from the entanglement of nuclear spin and molecule rotation/ vibration.

The broad NH_3_ resonance in the ^1^H NMR spectrum of NH_3_@**2** is associated with the quadrupolar relaxation of the ^14^N nucleus, and is interpreted in terms of the ^14^N spin lattice relaxation time: *T*_1_(^14^N)=2.66 ms. The scalar relaxation of the second kind mechanism is verified by broadening of the ^1^H linewidth with increasing temperature. A rotational correlation time of *τ_c_*=1.5 ps. is estimated for endohedral NH_3_. An experimentally determined rotational correlation time of *τ_c_*=57±5 ps. for the open fullerene **2** confirms free rotation of the encapsulated molecule.

The difference IR spectrum of NH_3_@**2** and **2** at 5 K, identified three vibrations of NH_3_ (*ν*_1_, *ν*_3_ and *ν*_4_) redshifted in comparison with free NH_3_. A rapid decrease in IR peak intensity with increasing temperature indicates the presence of a large number of excited translational/ rotational states which are populated above 50 K. The structures of these states is complex, so that no resolved IR peaks are observed. Nevertheless, rotational freedom is possible, in accordance with NMR observations.

## Experimental Section

### Synthesis and Characterization of Open‐Cage Fullerene (OCF) Derivatives

Reactions requiring dry conditions were conducted under an argon atmosphere using standard Schlenk and syringe techniques with freshly distilled solvents. All apparatus was dried in a hot in oven (>140 °C, 12 h) before being cooled in a sealed dessicator over silica gel or assembled while hot and cooled under vacuum (0.1 mm Hg). 1,2‐Dichlorobenzene was distilled from CaH_2_ at 55 °C under a vacuum of 15 mm Hg. Ethanol was dried over 3 Å molecular sieves. All other reagents, solvents or gases were used as received from commercial suppliers. High‐pressure reactions were conducted in a Parr^®^ pressure vessel of 75 mL volume and 1 inch i.d., sealed with PTFE or graphite gasket. High‐pressure reactions were heated using an external oil bath and temperature monitoring was conducted using an external thermostat. NMR spectra were recorded on Bruker AVII400, AVIIIHD400 or AVIIIHD500 FT‐NMR spectrometers in the indicated solvent at 298 K. ^1^H chemical shifts are reported as values in ppm referenced to residual solvent. Spectra collected in 1,2‐dichlorobenzene‐*d*_4_ are referenced to residual solvent at *δ*_H_=7.19 ppm, *δ*_H_=6.94 ppm; this solvent assignment is referenced to TMS (*δ*_H_=0 ppm). The following abbreviations are used to assign multiplicity and may be compounded: s=singlet, d=doublet, t=triplet, q=quartet and m=multiplet. Coupling constants, *J*, are measured in Hertz (Hz). ^13^C spectra are proton decoupled and referenced to solvent. ^13^C chemical shifts are reported to 2 d.p. in order to distinguish closely neighbouring resonances. Low‐resolution mass spectra were recorded using a MaXis mass spectrometer (Bruker Daltronics) equipped with Time of Flight (t.o.f.) analyzer using positive electrospray ionization. Samples were infused via a syringe driver at a constant flow rate of 3 μL min^−1^. High‐resolution mass spectra were obtained using a solariX FT‐ICR mass spectrometer equipped with a 4.7T superconducting magnet, using positive electrospray ionization. Values of *m*/*z* are reported in atomic mass units.

### Open Fullerene 1

Open fullerene **1** was prepared according to the method of Murata,^12^ our procedure differing only in purification which was carried out by column chromatography over SiO_2_ eluted with a gradient of 2 % → 5 % EtOAc in toluene. Spectroscopic data were consistent with the published data.

### CH_4_@1

A Parr^®^ pressure vessel equipped with glass reactor insert was charged with a solution of open fullerene **1** (305 mg, 0.27 mmol) in 1‐chloronaphthalene (15 mL of ≥85 % technical grade containing ≈10 % 2‐chloronaphthalene). The reactor vessel was sealed and flushed with CH_4_ before charging with CH_4_ to 101 atm at room temperature. The vessel was heated to 200 °C (external oil bath temperature) and stirred at this temperature with an internal pressure of 153 atm for 68 h, then cooled to room temperature and the pressure slowly released. The residue was diluted with 1‐chloronaphthalene (20 mL) and filtered through a short SiO_2_ column with CHCl_3_ (elutes 1‐chloronaphthalene near the solvent front) followed by EtOAc (elutes CH_4_@**1** near the solvent front). The EtOAc filtrate was concentrated in vacuo at room temperature to yield the title compound as a red/brown powder (310 mg of an inseparable 65:35 mixture of CH_4_@**1**: **1**, 100 % yield).

### Data for the CH_4_@1 Component of the Mixed NMR Spectra:

^1^H NMR (400 MHz, [D_8_]THF) *δ*_H_=7.72 (1 H, t, *J=*7.8 Hz), 7.62 (1 H, t, *J=*7.8 Hz), 7.32 (2 H, d, *J=*7.8 Hz), 7.21 (2 H, d, *J=*7.8 Hz), 7.02 (1 H, d, *J=*10.2 Hz), 6.30 (1 H, d, *J=*10.2 Hz), 1.23 (9 H, s), 1.12 (9 H, s), −12.43 ppm (encapsulated CH_4_, 4 H, s); ^13^C NMR (125.7 MHz, [D_8_]THF) *δ*_C_=190.68, 185.40, 182.11, 180.48, 169.23, 169.13, 164.99, 163.38, 156.73, 153.37, 152.21, 151.59, 151.18, 151.05, 150.84, 150.62, 150.55, 150.48, 150.35, 150.26, 149.96, 149.90, 149.85, 148.19, 147.09, 146.51, 146.47, 146.41, 145.96, 145.87, 145.80, 145.51, 144.71, 144.39, 144.31, 144.07, 143.54, 143.36, 142.73, 142.58, 142.15, 141.74, 140.46, 140.25, 139.78, 139.54, 139.27, 139.11, 138.87, 138.82, 138.79, 138.68, 138.45, 137.80, 137.74, 137.72, 137.61, 137.52, 136.21, 134.84, 134.60, 132.79, 132.30, 132.28, 129.97, 128.10, 126.58, 121.20, 120.77, 118.36, 118.19, 60.70, 55.62, 38.40, 38.37, 30.38, 30.27, −19.24 ppm. One overlapping resonance is not reported; ^1^H NMR (500 MHz, 1,2‐dichlorobenzene‐*d*_4_) *δ*_H_=−12.59 ppm (encapsulated CH_4_, 4 H, s), *T*_1_=7.53±0.03 s.; Proton‐coupled ^13^C NMR (125.7 MHz, 1,2‐dichlorobenzene‐*d*_4_) *δ*_C_=−19.47 ppm (encapsulated CH_4_, pentet, *J=*124.8 Hz); ES+ *m*/*z* 1151.20 (C_83_H_30_N_2_O_4_S (CH_4_@**1**) + H^+^).

### Data for the (Minor) Open Fullerene 1 Component of the Mixed ^1^H Spectrum:

^1^H NMR (400 MHz, [D_8_]THF) *δ*=7.72 (1 H, t, *J=*7.8 Hz), 7.61 (1 H, t, *J=*7.8 Hz), 7.32 (2 H, d, *J=*7.8 Hz), 7.20 (2 H, d, *J=*7.8 Hz), 7.04 (1 H, d, *J=*10.2 Hz), 6.30 (1 H, d, *J=*10.2 Hz), 1.23 (9 H, s), 1.11 ppm (9 H, s); ES+ *m*/*z* 1135.17 (C_82_H_26_N_2_O_4_S (**1**) + H^+^).

### NH_3_@2

Open fullerene **1** (26 mg, 0.023 mmol) was dried at 140 °C (external oil bath temperature) for 2 h under a vacuum of 0.3 mm Hg, before cooling under argon and addition of degassed 1,2‐dichlorobenzene (4 mL). The solution was cooled to 0 °C (ice/salt bath) and NH_3_ (33 μL of a 7 n solution in methanol, 0.23 mmol) was added drop‐wise. The resulting mixture was stirred at 0 °C for 10 min. before addition of NaBH_4_ (0.2 mL of a freshly prepared 58 mm solution in EtOH, 0.011 mmol) and stirring at 0 °C for 15 min. further. 1 m HCl (2 mL) was then added and the cooling bath removed. After warming to room temperature, the mixture was stirred overnight before separation of the organic phase and extraction of the aqueous phase with 1,2‐dichlorobenzene (1 mL). The combined organic extracts were filtered through a short SiO_2_ column with CHCl_3_ (elutes 1,2‐dichlorobenzene near the solvent front) followed by EtOAc (elutes NH_3_@**2** near the solvent front). The EtOAc filtrate was concentrated in vacuo. Purification by column chromatography (SiO_2_ eluted with 94:4:2 toluene:EtOAc:AcOH) gave the title compound as a brown/black solid (12 mg, 45 % yield, 92 % NH_3_ encapsulation).

### Data for the NH_3_@2 Component of the Mixed NMR Spectra:

^1^H NMR (500 MHz, 1,2‐dichlorobenzene‐*d*_4_) *δ*_H_=7.62 (1 H, t, *J=*7.9 Hz), 7.54 (1 H, t, *J=*7.9 Hz), 7.42 (1 H, d, *J=*4.6 Hz), 7.27–7.22 (3 H, m), 7.20–7.13 (2 H, m), 6.60 (1 H, d, *J=*10.3 Hz), 3.75, (1 H, d, *J=*4.6 Hz), 1.24 (9 H, s), 1.13 (9 H, s), −12.35 ppm (encapsulated NH_3_, 3 H, broad s, including approx. 0.3 % overall intensity d, *J=*59.8 Hz attributed to natural abundance ^15^NH_3_@**2)** ppm. Encapsulated NH_3_, *T*_1_=5.05±0.02 s.; ^13^C NMR (125.7 MHz, 1,2‐dichlorobenzene‐*d*_4_) *δ*_C_=198.04, 186.30, 183.26, 169.08, 169.03, 164.53, 163.35, 157.99, 157.05, 153.97, 153.58, 151.36, 151.24, 151.16, 151.09, 151.01, 150.84, 150.82, 150.45, 150.13, 150.11, 149.92, 149.61, 149.16, 148.87, 147.68, 147.08, 145.86, 145.54, 145.44, 145.36, 145.31, 144.28, 144.13, 144.05, 143.93, 143.53, 143.51, 142.65, 142.12, 141.96, 141.28, 141.27, 139.40, 138.83, 138.36, 138.26, 137.84, 137.70, 137.68, 137.66, 137.63, 137.56, 137.34, 137.27, 137.18, 136.90, 135.85, 135.14, 134.73, 134.00, 132.45, 131.31, 130.75, 130.39, 125.17, 120.52, 120.31, 118.00, 117.74, 83.08, 59.58, 55.01, 38.11, 38.08, 30.20, 30.13 ppm. Five overlapping resonances are not reported; ES+ *m*/*z* 1154.2103 (C_83_H_31_N_3_O_4_S (NH_3_@**2**) + H^+^).

Data for the minor component of the mixed ^1^H spectrum in 1,2‐dichlorobenzene‐*d*_4_ (“empty” open fullerene **2**) has spectroscopic data consistent with that published by Murata et al.^14^

### Measurement of ^1^H and ^13^C Spin‐Lattice Relaxation

Experimental ^1^H spin‐lattice relaxation curves were measured for CH_4_@**1** (17.4 mm) and NH_3_@**2** (17.6 mm) in degassed 1,2‐dichlorobenzene‐*d*_4_, and the experimental ^13^C spin‐lattice relaxation curve was measured for the C(H)OH methine carbon on the orifice of NH_3_@**2** (7.3 mm) in degassed CDCl_3_. All spectra were acquired at 11.7 T and 25 °C, using a Bruker AVIIIHD500 FT‐NMR spectrometer. Spin‐lattice relaxation times *T*_1_ were estimated using the saturation‐recovery pulse sequence. The 90° pulse was calibrated for each sample using Bruker TopSpin and the saturation‐recovery sequence employed a 16‐point delay list (0.01, 0.05, 0.1, 0.2, 0.3, 0.5, 0.75, 1, 1.5, 2, 2.5, 5, 7.5, 10, 15, 30 s.). Signals of interest from the saturation‐recovery experiments were integrated using Bruker TopSpin, and the data were fitted using Mathematica. Signal amplitudes was normalized to the last data point. The fitted curves have a single‐exponential form.

### IR Spectroscopy

Samples of NH_3_@**2** and H_2_O@**2** with filling factors of *f*=0.9 (NH_3_@**2**) and 0.35 (H_2_O@**2**) were studied. The fraction of empty cages is 1−*f*. The sample of NH_3_@**2** contained a small amount of H_2_O@**2** so the resonance lines specific to NH_3_ were identified by comparing the NH_3_@**2** and H_2_O@**2** spectra. Some spectral regions were opaque because of the absorption by the open fullerene. The samples were pressed into pellets of 3 mm diameter and of thickness *d=*155 μm (NH_3_@**2)** and 190 μm (H_2_O@**2)**. Spectra were recorded with a Vertex 80v (Bruker Optics) spectrometer between 600 and 9000 cm^−1^ with a liquid nitrogen cooled HgCdTe detector. Sample temperature was controlled between 5 and 300 K with a continuous flow cryostat. Transmitted intensity through the sample, *I*_s_, was referenced to the intensity through a 3 mm diameter hole, *I*_0_. The absorption coefficient α was calculated from the ratio *Tr*=*I*_s_/*I*_0_ as *α*=−*d* ^−1^ln[*Tr*(1−*R*)^−2^] where the factor (1−*R* )^2^ with *R*=(*η*−1)^2^(*η*+1)^−2^ corrects for two back reflections, one from the sample front and one from the back face. We used *η*=2 of solid C_60_ as the refraction index.^41^ The spectra in Figures 9 and 10 are difference spectra α(*T*)−α(300 K) with the base line removed.

### Inelastic Neutron Scattering Spectroscopy

INS was performed at the high‐flux reactor source of the Institut Laue‐Langevin, Grenoble. Three spectrometers were employed; IN4c and IN6 are time‐of‐flight (t.o.f.) INS spectrometers and IN1‐Lagrange is a triple‐axis spectrometer equipped with a large area graphite analyser. IN4c and IN1‐Lagrange have been described in earlier papers24a,24b IN6 is designed for quasi‐elastic and inelastic neutron scattering. It operates on a cold neutron source and provides good resolution at low energy transfer, accessing both neutron energy (NE) gain and NE loss components of the spectrum. By convention the NE gain is defined with negative values of energy transfer Δ*E*. The powdered samples were wrapped in Al foil sachets for mounting in the cryostat. A “blank” sample of open fullerene (**1**) with identical mass to that of the CH_4_@**1** sample was employed. In order to remove spectral features arising from fullerene cage modes and scattering from the construction materials of the cryostat, the INS spectra from the “blank” were subtracted from those recorded on the sample containing CH_4_. The spectra were recorded at cryogenic temperatures and the data is openly available [http://doi.ill.fr/10.5291/ILL‐DATA.7‐04‐148].

### Computational Experiments

Computational experiments were carried out using the Gaussian 09 software package.^42^ A model structure (**1 b**) for open fullerene **1** in which the 6‐*tert*‐butyl pyridyl substituents were replaced by methyl substituents, was used. Structures and transition states were optimised using DFT with the M06‐2X functional^43^ and Dunning's correlation consistent basis set cc‐pVDZ.^44^ Frequency calculations were carried out for each stationary point to check that the optimised geometry corresponded to a minimum or a transition state, and to allow the Gibbs free energies and entropies to be calculated at defined temperatures and pressures using the Gaussian freqchk utility. Vibrations were not scaled and low frequency vibrations were not removed. Electronic energies were calculated at the above geometries using M06‐2X with the cc‐pVTZ basis set and were corrected for basis set superposition errors using the counterpoise method.^45^

## Conflict of interest

*The authors declare no conflict of interest*.

## Supporting information

As a service to our authors and readers, this journal provides supporting information supplied by the authors. Such materials are peer reviewed and may be re‐organized for online delivery, but are not copy‐edited or typeset. Technical support issues arising from supporting information (other than missing files) should be addressed to the authors.

Supplementary

## References

[1] H. W. Kroto , J. R. Heath , S. C. O'Brien , R. F. Curl , R. E. Smalley , Nature 1985, 318, 162–163.

[2] M. H. Levitt , Philos. Trans. R. Soc. A 2013, 371, 20120429. 10.1098/rsta.2012.0429 23918717

[3a] X. Lu , L. Feng , T. Akasaka , S. Nagase , Chem. Soc. Rev. 2012, 41, 7723–7760; 22907208 10.1039/c2cs35214a

[3b] A. A. Popov , S. Yang , L. Dunsch , Chem. Rev. 2013, 113, 5989–6113. 23635015 10.1021/cr300297r

[4] S. Osuna , M. Swart , M. Sola , Chem. Eur. J. 2009, 15, 13111–13123. 19859923 10.1002/chem.200901224

[5] T. A. Murphy , T. Pawlik , A. Weidinger , M. Hohne , R. Alcala , J. M. Spaeth , Phys. Rev. Lett. 1996, 77, 1075–1078. 10062984 10.1103/PhysRevLett.77.1075

[6] Y. Rubin , Top. Curr. Chem. 1999, 199, 67–91.

[7a] K. Komatsu , M. Murata , Y. Murata , Science 2005, 307, 238–240; 15653499 10.1126/science.1106185

[7b] M. Murata , S. Maeda , Y. Morinaka , Y. Murata , K. Komatsu , J. Am. Chem. Soc. 2008, 130, 15800–15801. 18959401 10.1021/ja8076846

[8a] K. Kurotobi , Y. Murata , Science 2011, 333, 613–616; 21798946 10.1126/science.1206376

[8b] A. Krachmalnicoff , M. H. Levitt , R. J. Whitby , Chem. Commun. 2014, 50, 13037–13040; 10.1039/c4cc06198e 25228024

[8c] R. Zhang , M. Murata , T. Aharen , A. Wakamiya , T. Shimoaka , T. Hasegawa , Y. Murata , Nat. Chem. 2016, 8, 435–441. 27112631 10.1038/nchem.2464

[9a] A. Krachmalnicoff , R. Bounds , S. Mamone , S. Alom , M. Concistre , B. Meier , K. Kouril , M. E. Light , M. R. Johnson , S. Rols , A. J. Horsewill , A. Shugai , U. Nagel , T. Room , M. Carravetta , M. H. Levitt , R. J. Whitby , Nat. Chem. 2016, 8, 953–957; 27657872 10.1038/nchem.2563

[9b] R. Zhang , M. Murata , A. Wakamiya , T. Shimoaka , T. Hasegawa , Y. Murata , Sci. Adv. 2017, 3, e1602833. 10.1126/sciadv.1602833 PMC5400425 28439559

[10a] C. Beduz , M. Carravetta , J. Y. C. Chen , M. Concistre , M. Denning , M. Frunzi , A. J. Horsewill , O. G. Johannessen , R. Lawler , X. Lei , M. H. Levitt , Y. Li , S. Mamone , Y. Murata , U. Nagel , T. Nishida , J. Ollivier , S. Rols , T. Rõõm , R. Sarkar , N. J. Turro , Y. Yang , Proc. Natl. Acad. Sci. USA 2012, 109, 12894–12898; 22837402 10.1073/pnas.1210790109 PMC3420201

[10b] S. Mamone , M. Concistre , E. Carignani , B. Meier , A. Krachmalnicoff , O. G. Johannessen , X. Lei , Y. Li , M. Denning , M. Carravetta , K. Goh , A. J. Horsewill , R. J. Whitby , M. H. Levitt , J. Chem. Phys. 2014, 140, 194306; 24852537 10.1063/1.4873343

[10c] B. Meier , S. Mamone , M. Concistrè , J. Alonso-Valdesueiro , A. Krachmalnicoff , R. J. Whitby , M. H. Levitt , Nat. Commun. 2015, 6, 8112. 26299447 10.1038/ncomms9112 PMC4560827

[11] T. Futagoishi , M. Murata , A. Wakamiya , Y. Murata , Angew. Chem. Int. Ed. 2015, 54, 14791–14794; 10.1002/anie.201507785 26473764

[12] T. Futagoishi , M. Murata , A. Wakamiya , T. Sasamori , Y. Murata , Org. Lett. 2013, 15, 2750–2753. 23675898 10.1021/ol401083c

[13] M. Stanisky , R. J. Cross , M. Saunders , J. Am. Chem. Soc. 2009, 131, 3392–3395. 19209931 10.1021/ja809831a

[14] T. Futagoishi , T. Aharen , T. Kato , A. Kato , T. Ihara , T. Tada , M. Murata , A. Wakamiya , H. Kageyama , Y. Kanemitsu , Y. Murata , Angew. Chem. Int. Ed. 2017, 56, 4261–4265; 10.1002/anie.201701212 28300342

[15a] S. Iwamatsu , C. M. Stanisky , R. J. Cross , M. Saunders , N. Mizorogi , S. Nagase , S. Murata , Angew. Chem. Int. Ed. 2006, 45, 5337–5340; 10.1002/anie.200601241 16847866

[15b] L. J. Shi , D. Z. Yang , F. Colombo , Y. M. Yu , W. X. Zhang , L. B. Gan , Chem. Eur. J. 2013, 19, 16545–16549. 24281804 10.1002/chem.201303501

[16] K. E. Whitener, Jr. , M. Frunzi , S. Iwamatsu , S. Murata , R. J. Cross , M. Saunders , J. Am. Chem. Soc. 2008, 130, 13996–13999. 18817388 10.1021/ja805579m

[17] K. E. Whitener, Jr. , R. J. Cross , M. Saunders , S. Iwamatsu , S. Murata , N. Mizorogi , S. Nagase , J. Am. Chem. Soc. 2009, 131, 6338–6339. 19368384 10.1021/ja901383r

[18] T. Futagoishi , M. Murata , A. Wakamiya , Y. Murata , Angew. Chem. Int. Ed. 2017, 56, 2758–2762; 10.1002/anie.201611903 28139014

[19] C. S. Chen , T. S. Kuo , W. Y. Yeh , Chem. Eur. J. 2016, 22, 8773–8776. 27123778 10.1002/chem.201601737

[20] C. S. Chen , W. Y. Yeh , Chem. Eur. J. 2016, 22, 16425–16428. 27616427 10.1002/chem.201604114

[21a] G. C. Vougioukalakis , M. M. Roubelakis , M. Orfanopoulos , Chem. Soc. Rev. 2010, 39, 817–844; 20111794 10.1039/b913766a

[21b] S. Iwamatsu , S. Murata , Synlett 2005, 2117–2129;

[21c] L. Gan , D. Yang , Q. Zhang , H. Huang , Adv. Mater. 2010, 22, 1498–1507. 20437499 10.1002/adma.200903705

[22] A. Antušek , K. Jackowski , M. Jaszunski , W. Makulski , M. Wilczek , Chem. Phys. Lett. 2005, 411, 111–116.

[23] B. Bennett , W. T. Raynes , Mol. Phys. 1987, 61, 1423–1430.

[24a] S. Mamone , M. Jimenez-Ruiz , M. R. Johnson , S. Rols , A. J. Horsewill , Phys. Chem. Chem. Phys. 2016, 18, 29369–29380; 27735010 10.1039/c6cp06059e

[24b] K. S. K. Goh , M. Jimenez-Ruiz , M. R. Johnson , S. Rols , J. Ollivier , M. S. Denning , S. Mamone , M. H. Levitt , X. Lei , Y. Li , N. J. Turro , Y. Murata , A. J. Horsewill , Phys. Chem. Chem. Phys. 2014, 16, 21330–21339; 25178254 10.1039/c4cp03272a

[24c] S. Mamone , M. R. Johnson , J. Ollivier , S. Rols , M. H. Levitt , A. J. Horsewill , Phys. Chem. Chem. Phys. 2016, 18, 1998–2005; 26687060 10.1039/c5cp07146a

[24d] A. J. Horsewill , K. S. Panesar , S. Rols , J. Ollivier , M. R. Johnson , M. Carravetta , S. Mamone , M. H. Levitt , Y. Murata , K. Komatsu , J. Y. C. Chen , J. A. Johnson , X. Lei , N. J. Turro , Phys. Rev. B 2012, 85, 205440.

[25] A. J. Horsewill , K. S. Panesar , S. Rols , M. R. Johnson , Y. Murata , K. Komatsu , S. Mamone , A. Danquigny , F. Cuda , S. Maltsev , M. C. Grossel , M. Carravetta , M. H. Levitt , Phys. Rev. Lett. 2009, 102, 013001. 19257185 10.1103/PhysRevLett.102.013001

[26] G. H. Kwei , F. Trouw , B. Morosin , H. F. King , J. Chem. Phys. 2000, 113, 320–328.

[27a] M. Prager , W. Langel , J. Chem. Phys. 1988, 88, 7995–7999;

[27b] M. Prager , W. Langel , J. Chem. Phys. 1989, 90, 5889–5890;

[27c] W. Press , Single-Particle Rotations in Molecular Crystals, Springer, Berlin, Heidelberg, New York, 1981.

[28] P. M. Felker , Z. Bacic , J. Chem. Phys. 2016, 144, 201101. 27250272 10.1063/1.4953180

[29a] W. Press , A. Kollmar , Solid State Commun. 1975, 17, 405–408;

[29b] H. Glättli , A. Sentz , M. Eisenkremer , Phys. Rev. Lett. 1972, 28, 871–873;

[29c] F. H. Frayer , G. E. Ewing , J. Chem. Phys. 1967, 46, 1994–1995;

[29d] F. H. Frayer , G. E. Ewing , J. Chem. Phys. 1968, 48, 781–792;

[29e] K. J. Lushington , J. A. Morrison , Can. J. Phys. 1977, 55, 1580–1588.

[30a] L. G. Werbelow , J. Kowalewski , J. Chem. Phys. 1997, 107, 2775–2781;

[30b] R. K. Harris , N. C. Pyper , Mol. Phys. 1975, 29, 205–223;

[30c] N. C. Pyper , Mol. Phys. 1971, 21, 1–33;

[30d] N. C. Pyper , Mol. Phys. 1971, 22, 433–458;

[30e] N. C. Pyper , Mol. Phys. 1971, 21, 961–976;

[30f] N. C. Pyper , Mol. Phys. 1970, 19, 161–167.

[31] SpinDynamica code for Mathematica, programmed by Malcolm H. Levitt, with contributions by Jyrki Rantaharju, Andreas Brinkmann, and Soumya Singha Roy, available at: www.spindynamica.soton.ac.uk.

[32] C. Bengs, M. H. Levitt, *Magn. Reson. Chem* **2017**, https://doi.org/10.1002/mrc.4642. 10.1002/mrc.4642 PMC6001486 28809056

[33] A. Jerschow , Prog. Nucl. Magn. Reson. Spectrosc. 2005, 46, 63–78.

[34] J. Kowalewski , L. Maler , Nuclear Spin Relaxation in Liquids: Theory, Experiments, and Applications, CRC Press/Taylor & Francis, Boca Raton, FL 2006.

[35a] S. G. Kukolich , S. C. Wofsy , J. Chem. Phys. 1970, 52, 5477–5481;

[35b] J. T. Hougen , J. Chem. Phys. 1972, 57, 4207–4217.

[36a] W. D. White , R. S. Drago , J. Chem. Phys. 1970, 52, 4717–4723;

[36b] E. S. Laws , R. M. Stevens , W. N. Lipscomb , J. Chem. Phys. 1972, 56, 2029–2033;

[36c] S. P. Gejji , S. Lunell , J. Phys. Chem. 1990, 94, 4447–4449;

[36d] E. Kochanski , J. M. Lehn , B. Levy , Theor. Chim. Acta. 1971, 22, 111–129.

[37] R. D. Johnson , C. S. Yannoni , H. C. Dorn , J. R. Salem , D. S. Bethune , Science 1992, 255, 1235–1238. 17816831 10.1126/science.255.5049.1235

[38] M. S. Dresselhaus , G. Dresselhaus , A. Jorio , Group Theory: Applications to the Physics of Condensed Matter, Springer, Berlin, Heidelberg, 2008.

[39] “Infrared and Raman Spectra of Polyatomic Molecules”: G. Herzberg , Molecular Spectra and Molecular Structure , Vol. II , 1st ed., D. Van Nostrand Company, Inc., **1954**.

[40] C. Cumming , Can. J. Phys. 1955, 33, 635–639.

[41] C. C. Homes , P. J. Horoyski , M. L. W. Thewalt , B. P. Clayman , Phys. Rev. B 1994, 49, 7052–7055. 10.1103/physrevb.49.7052 10009437

[42] Gaussian 09, Revision D.01, M. J. Frisch, G. W. Trucks, H. B. Schlegel, G. E. Scuseria, M. A. Robb, J. R. Cheeseman, G. Scalmani, V. Barone, B. Mennucci, G. A. Petersson, H. Nakatsuji, M. Caricato, X. Li, H. P. Hratchian, A. F. Izmaylov, J. Bloino, G. Zheng, J. L. Sonnenberg, M. Hada, M. Ehara, K. Toyota, R. Fukuda, J. Hasegawa, M. Ishida, T. Nakajima, Y. Honda, O. Kitao, H. Nakai, T. Vreven, J. A. Montgomery, Jr., J. E. Peralta, F. Ogliaro, M. Bearpark, J. J. Heyd, E. Brothers, K. N. Kudin, V. N. Staroverov, T. Keith, R. Kobayashi, J. Normand, K. Raghavachari, A. Rendell, J. C. Burant, S. S. Iyengar, J. Tomasi, M. Cossi, N. Rega, J. M. Millam, M. Klene, J. E. Knox, J. B. Cross, V. Bakken, C. Adamo, J. Jaramillo, R. Gomperts, R. E. Stratmann, O. Yazyev, A. J. Austin, R. Cammi, C. Pomelli, J. W. Ochterski, R. L. Martin, K. Morokuma, V. G. Zakrzewski, G. A. Voth, P. Salvador, J. J. Dannenberg, S. Dapprich, A. D. Daniels, O. Farkas, J. B. Foresman, J. V. Ortiz, J. Cioslowski, and D. J. Fox, Gaussian, Inc., Wallingford CT, **2013**.

[43] Y. Zhao , D. G. Truhlar , Theor. Chem. Acc. 2008, 120, 215–241.

[44] T. H. Dunning, Jr. , J. Chem. Phys. 1989, 90, 1007–1023.

[45] S. F. Boys , F. Bernardi , Mol. Phys. 1970, 19, 553–566.

