# Supplementary material for: Synthesis and Properties of Open Fullerenes Encapsulating Ammonia and Methane
Source: Chemphyschem. 2018 Jan 4;19(3):266–76. doi: 10.1002/cphc.201701212 (PMC5838534; doi:10.1002/cphc.201701212)
Supplement: Supplementary file 1 — Supplementary [file CPHC-19-266-s001.pdf]

CHEMPHYSCHEM

Supporting Information

Synthesis and Properties of Open Fullerenes Encapsulating Ammonia and Methane

Sally Bloodworth,[a] John Gräsvik,[a] Shamim Alom,[a] Karel Kouřil,[a] Stuart J. Elliott,[a] Neil J. Wells,[a] Anthony J. Horsewill,[b] Salvatore Mamone,[b] Mónica Jiménez-Ruiz,[d] Stéphane Rols,[d] Urmas Nagel,[c] Toomas Rõõm,[c] Malcolm H. Levitt,[a] and Richard J. Whitby*[a]

cphc_201701212_sm_miscellaneous_information.pdf

CONTENTS

1 Characterisation of CH_4@**1** and NH_3@**2** S2

1.1 CH_4@**1** S2

1.2 NH_3@**2** S4

2 Kinetic Study of CH_4@**1** Dissociation S6

2.1 General Methods S6

2.2 Rate Constant Plots S6

3 Measurement of spin-lattice relaxation (T_1) S7

3.1 General Methods S7

3.2 Experimental spin-lattice relaxation curves S7

4 IR spectra of CH_4@**1** and NH_3@**2** S8

5 DFT calculations using Gaussian 09 S11

6 References S18

1. Synthesis and Characterisation of CH_4@1 and NH_3@2

1.1 CH_4@1

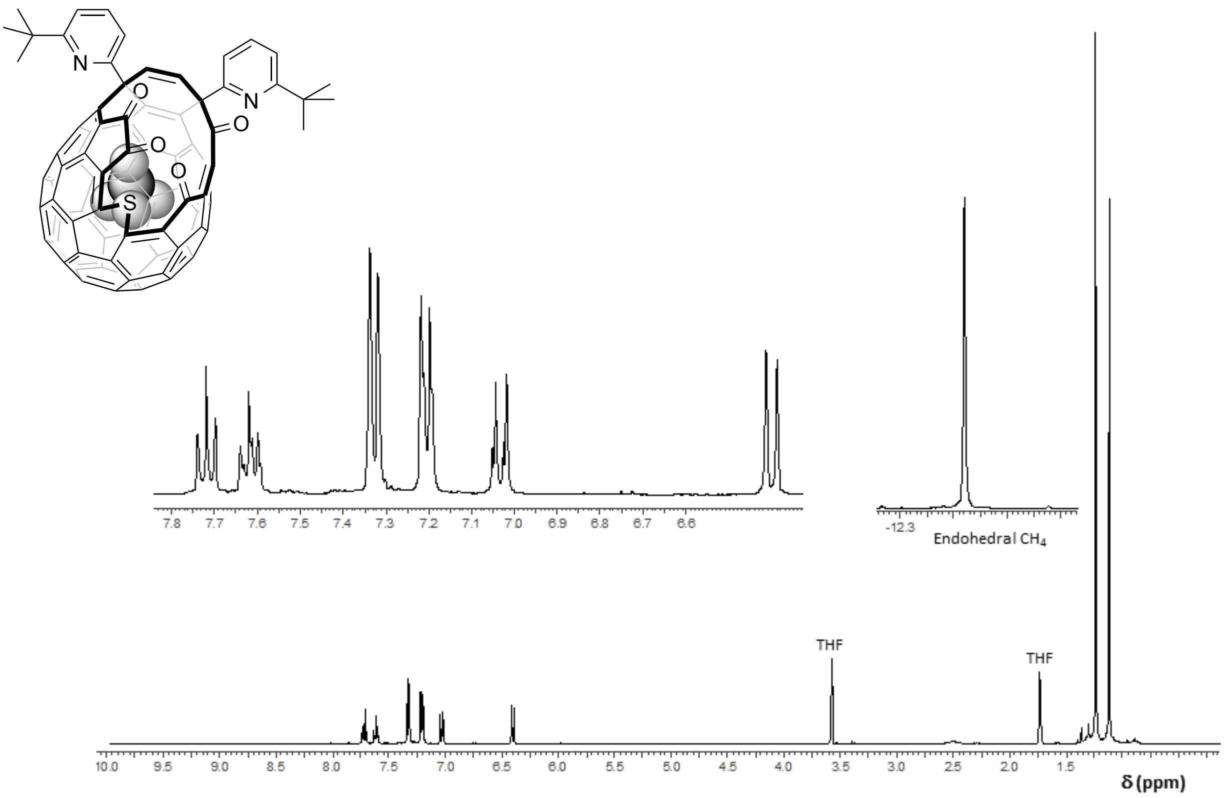

Figure **S1.0** Mixed component ^{1}H spectrum of CH_4@**1** and **1** (400 MHz, THF-d_8).

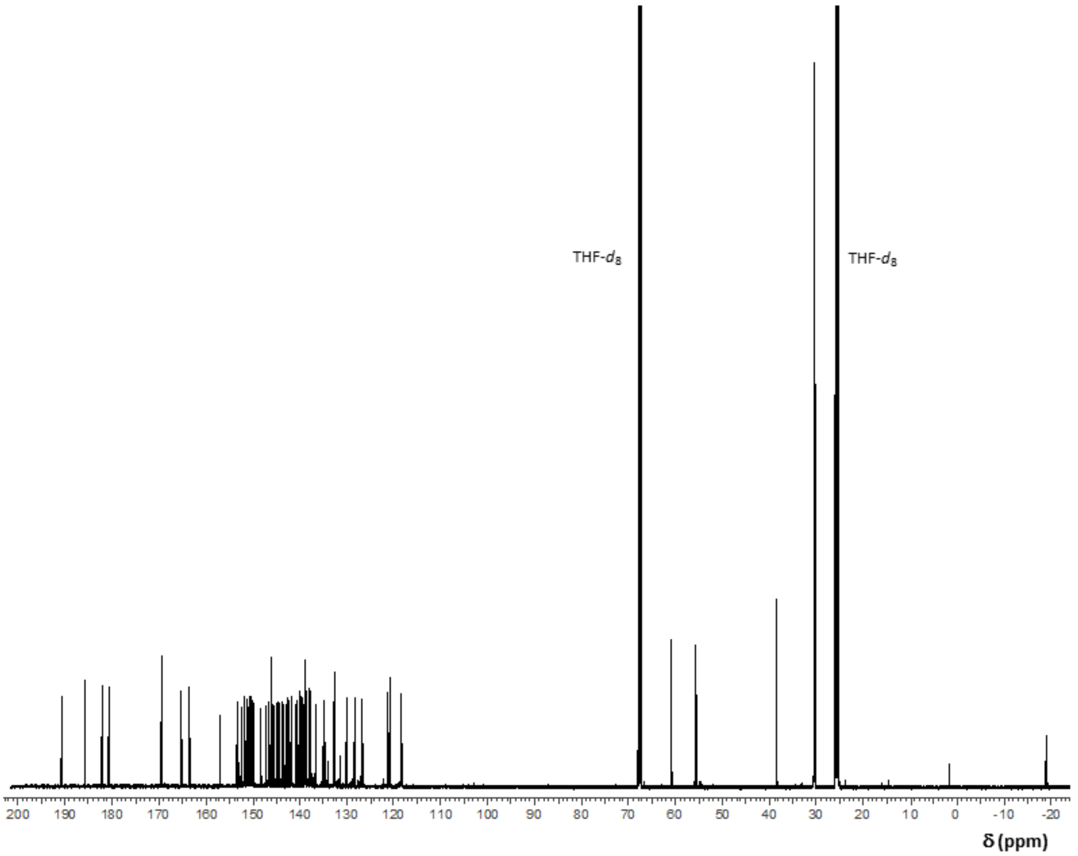

Figure **S1.1** Mixed component ^{13}C spectrum of CH_4@**1** and **1** (125.7 MHz, THF-d_8).

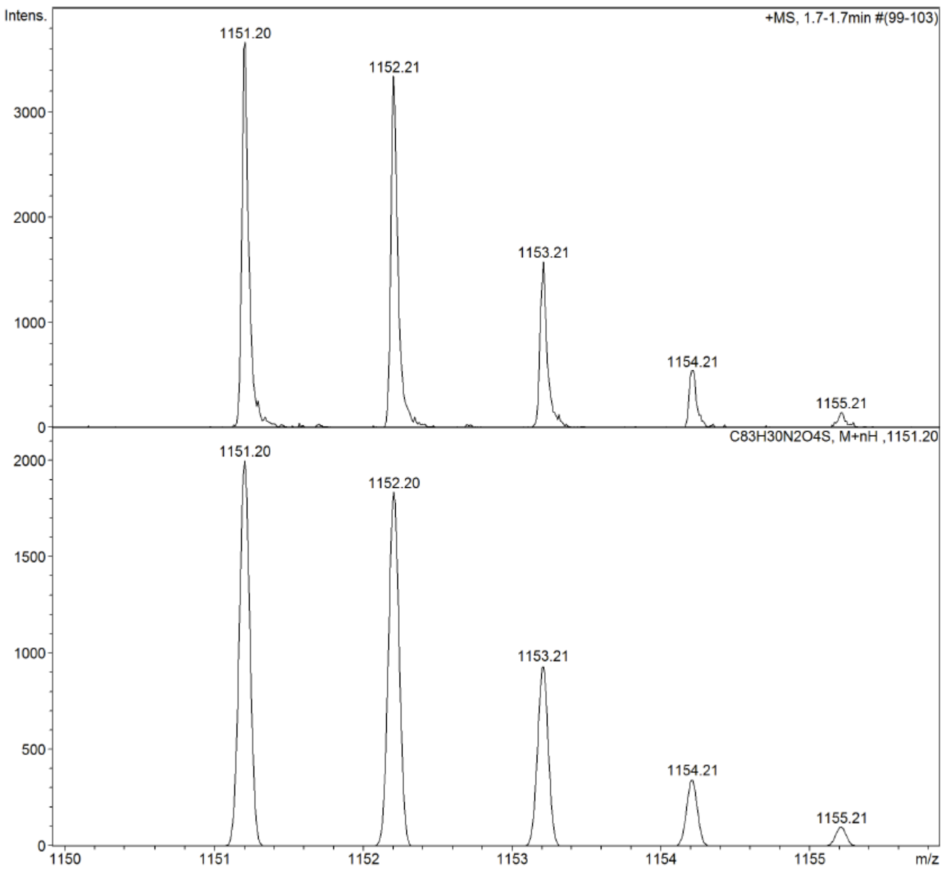

Figure **S1.2** ES+ *m/z* isotope patterns for $C_{83}H_{30}N_2O_4S$ (CH_4@**1**) + H^+, measured (top) and calculated (bottom).

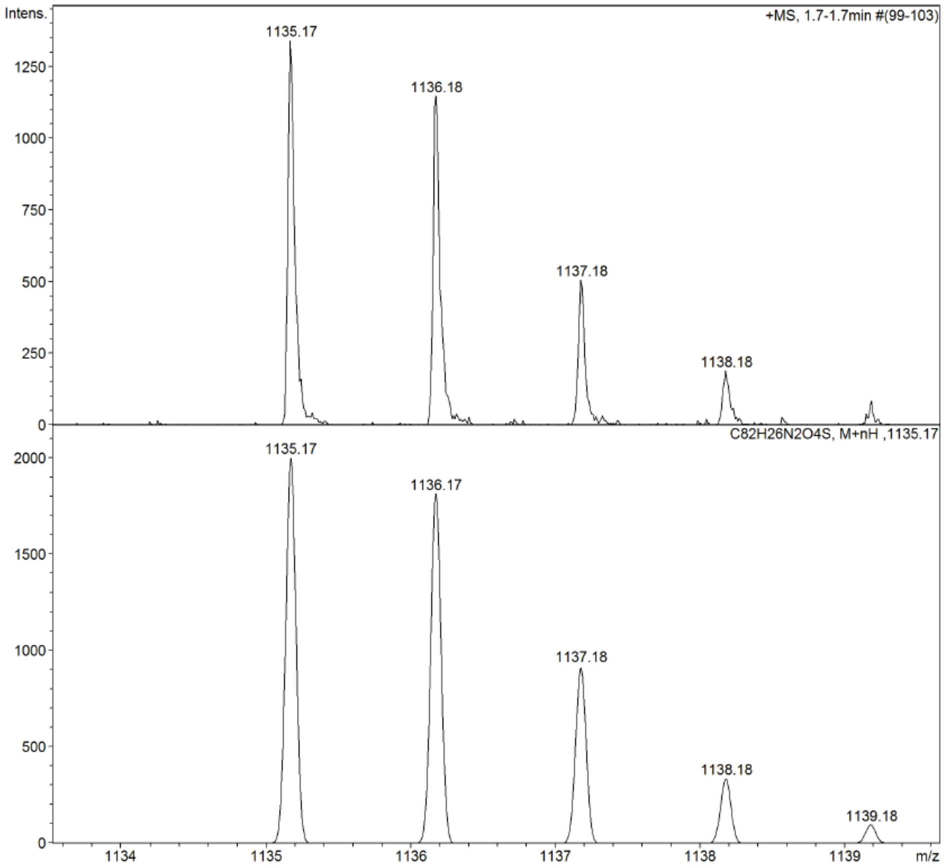

Figure **S1.3** ES+ *m/z* isotope patterns for $C_{82}H_{26}N_2O_4S$ (**1**) + H^+, measured (top) and calculated (bottom).

1.2 NH_3@2

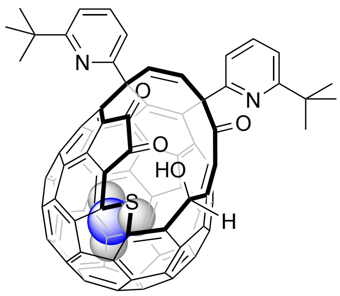

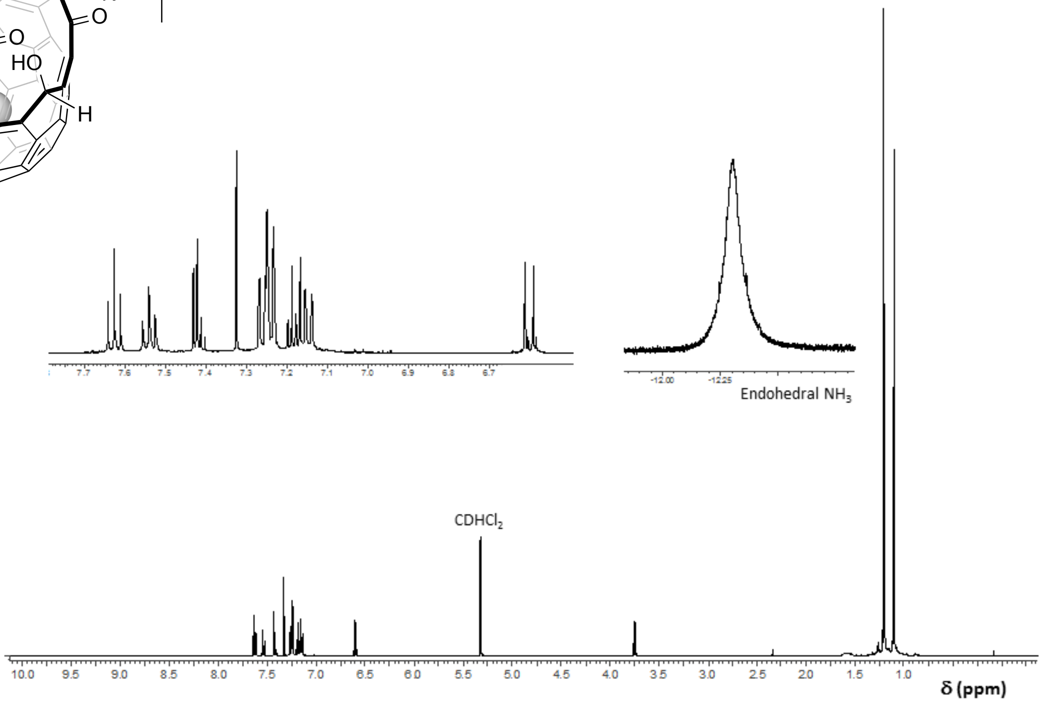

Figure **S1.4** Mixed component ^{1}H spectrum of NH_3@**2** and **2** (500 MHz, 1,2-dichlorobenzene-d_4).

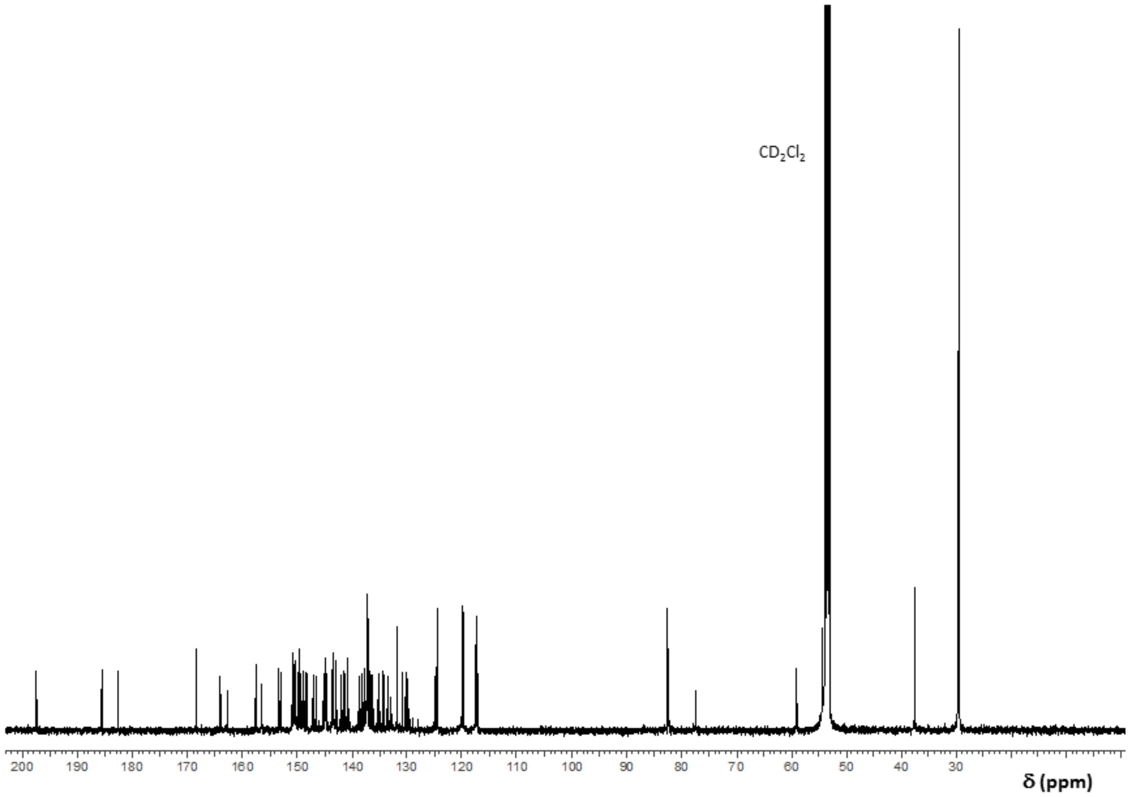

Figure **S1.5** Mixed component ^{13}C spectrum of NH_3@**2** and **2** (125.7 MHz, 1,2-dichlorobenzene-d_4).

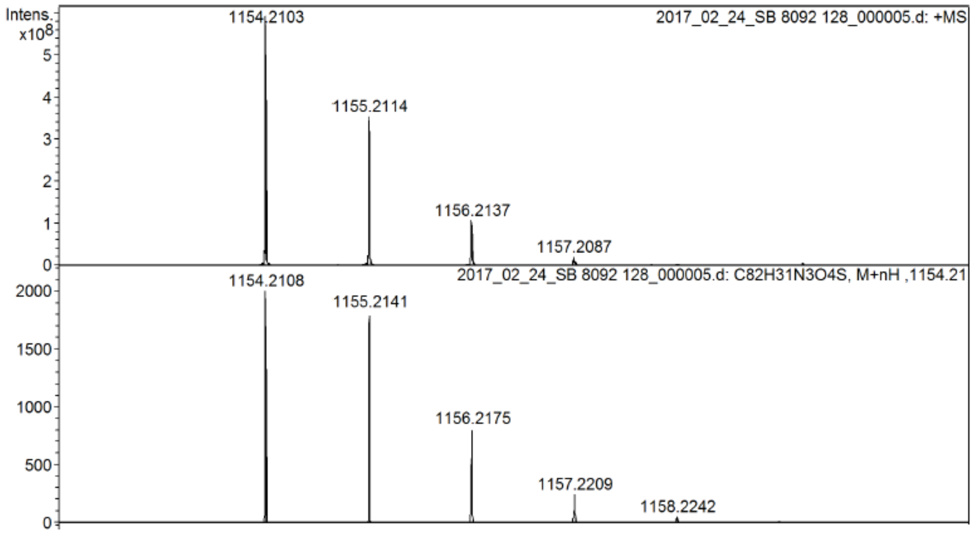

Figure **S1.6** High resolution ES+ *m/z* isotope patterns for $C_{82}H_{31}N_3O_4S$ (NH_3@**2**) + H^+, measured (top) and calculated (bottom).

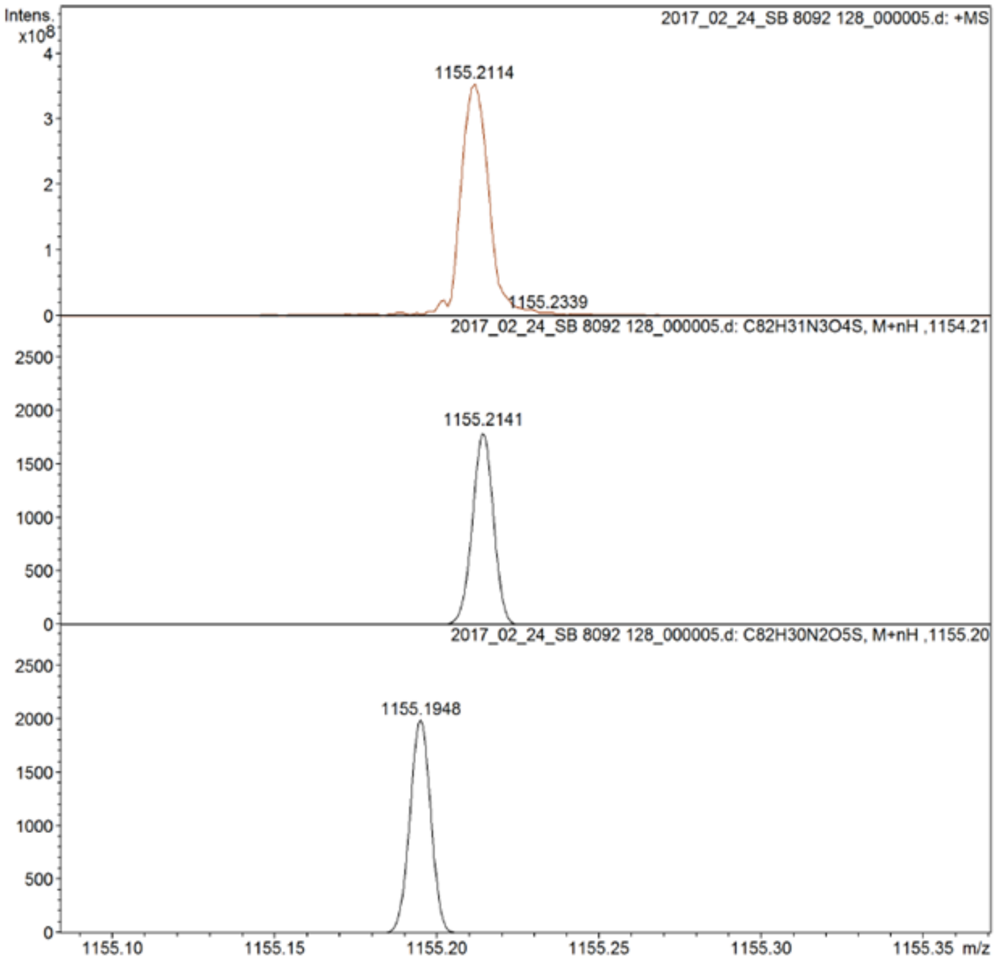

Figure **S1.7** Expansion of high resolution ES+ *m/z* isotope peak $^{13}C^{12}C_{81}H_{31}N_3O_4S$ (NH_3@**2** isotope) + H^+.
Measured (top) and calculated (middle).
Bottom: calculated ES+ *m/z* for $C_{82}H_{30}N_2O_5S$ (H_2O@**2**) + H^+. There is no overlap with $^{13}C^{12}C_{81}H_{31}N_3O_4S$ (NH_3@**2** isotope) + H^+, indicating that H_2O@**2** is not present in the measured data.

2. Kinetic Study of CH_4@1 Dissociation

2.1 General Methods

^{1}H NMR spectra of CH_4@**1** were recorded at 400 MHz in 1,2-dichlorobenzene-d_4 with a pulse delay d1 = 30 s. and are referenced to residual solvent at δ = 7.19 ppm and δ = 6.94 ppm. This solvent assignment is referenced to TMS (δ = 0 ppm). In plotting ln([CH_4@**1**]/[CH_4@**1**]$_0$) it is assumed that [CH_4@**1** + **1**] ≡ [CH_4@**1**]$_0$. Individual rate constants for thermal dissociation were determined from the peak integrals at δ = -12.59 ppm (CH_4@**1** endohedral resonance) and δ = 6.39 – 6.45 ppm (overlapping orifice alkene proton of CH_4@**1** +**1**). Estimated error in each rate constant is determined from the standard deviation of peak integrals. Estimated errors in E_a, $\Delta G^{\ddagger}$ $\Delta S^{\ddagger}$ and $\Delta H^{\ddagger}$ are determined from accumulation of the maximum error in each rate constant, and include an assumption of a standard deviation in temperature of ± 0.1 K.

2.2 Rate Constant Plots

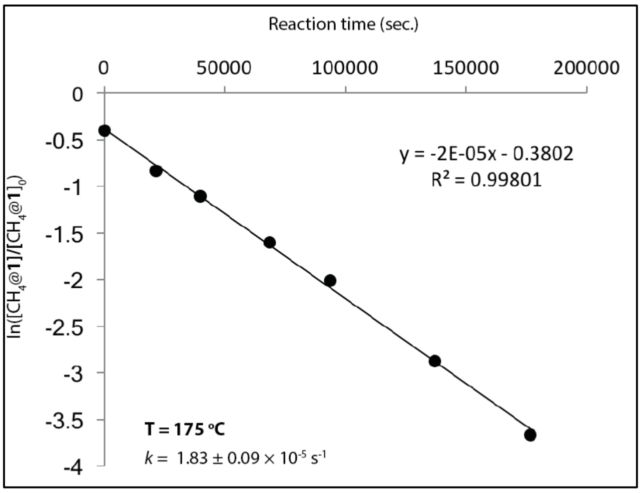

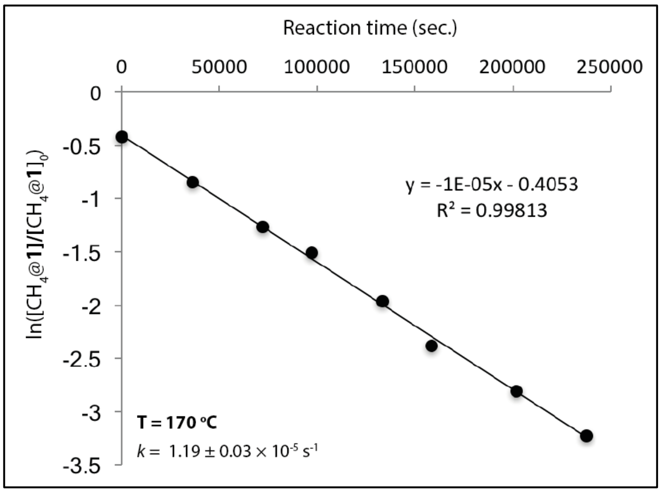

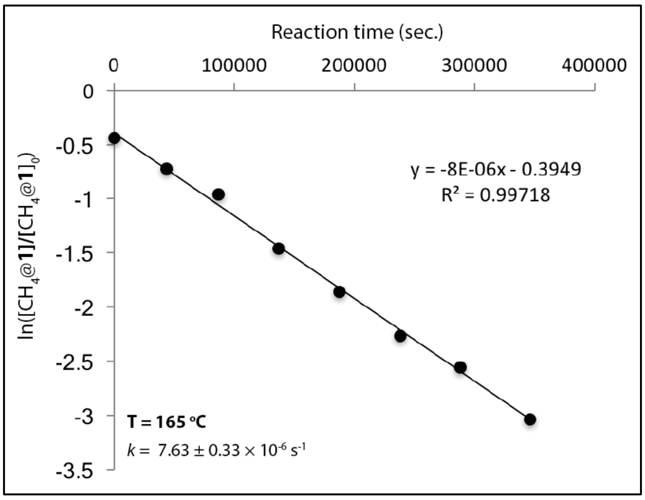

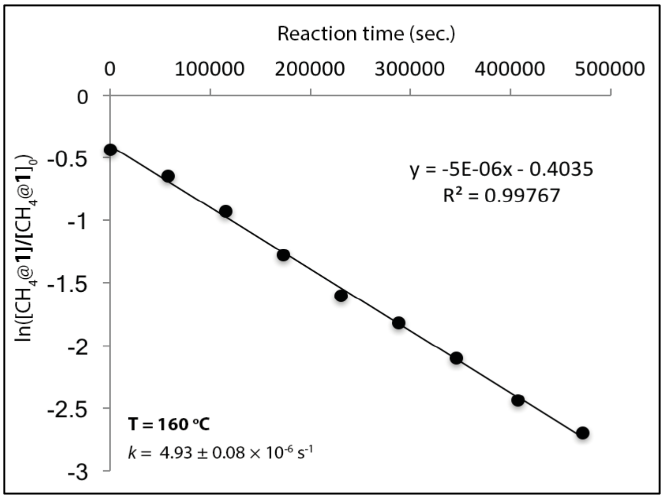

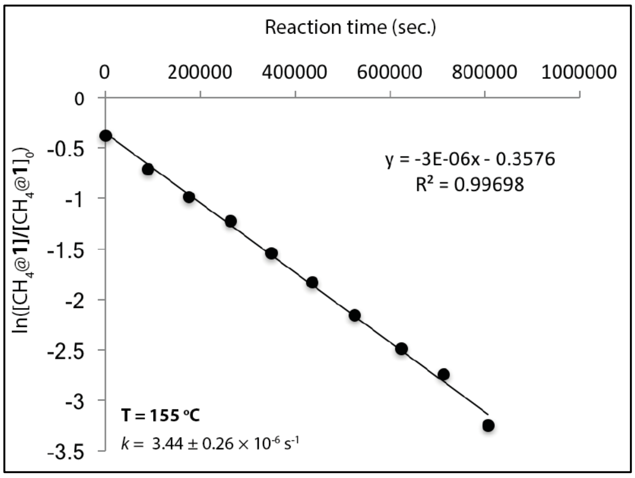

3. Measurement of spin-lattice relaxation

3.1 General methods

Experimental ^{1}H spin-lattice relaxation curves were measured for CH_4@**1** (17.4 mM) and NH_3@**2** (17.6 mM) in degassed 1,2-dichlorobenzene-d_4, and the experimental ^{13}C spin-lattice relaxation curve was measured for the C(H)OH methine carbon on the orifice of NH_3@**2** (7.3 mM) in degassed $CDCl_3$. All spectra were acquired at 11.7 T and 25 °C, using a Bruker AVIIIHD500 FT-NMR spectrometer. Spin-lattice relaxation times T_1 were estimated using the saturation-recovery pulse sequence. The 90° pulse was calibrated for each sample using Bruker TopSpin and the saturation-recovery sequence employed a 16-point delay list (0.01, 0.05, 0.1, 0.2, 0.3, 0.5, 0.75, 1, 1.5, 2, 2.5, 5, 7.5, 10, 15, 30 s.). Signals of interest from the saturation-recovery experiments were integrated using Bruker TopSpin, and the data were fitted using Mathematica. Signal amplitudes was normalized to the last data point. The fitted curves have a single-exponential form.

3.2 Experimental spin-lattice relaxation curves

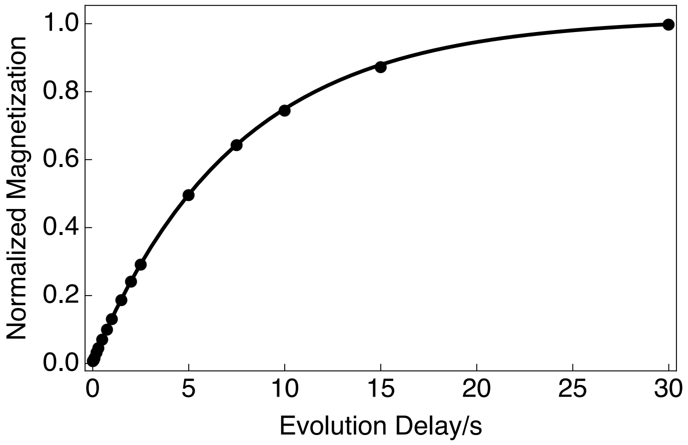

Figure **S3.1** Experimental ^{1}H spin-lattice relaxation curve for the encapsulated molecule in CH_4@**1** (17.4 mM), in 1,2-dichlorobenzene-d_4 solution at 11.7 T and 25 °C, measured by saturation recovery. T_1 = 7.53 ± 0.03 s.

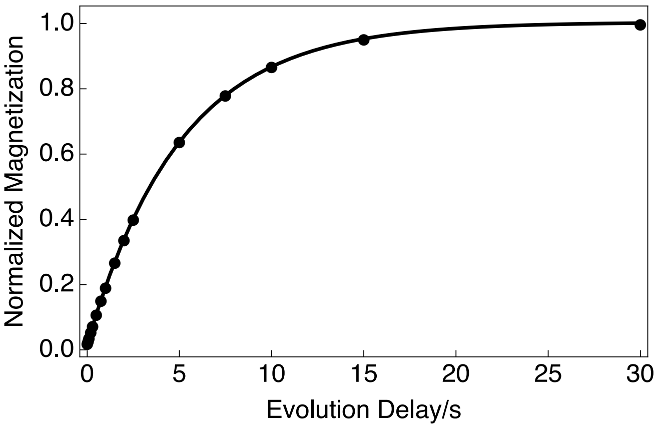

Figure **S3.2** Experimental ^{1}H spin-lattice relaxation curve for the encapsulated molecule in NH_3@**2** (17.6 mM), in 1,2-dichlorobenzene-d_4 solution at 11.7 T and 25 °C, measured by saturation recovery. $T_1 = 5.05 \pm 0.02$ s.

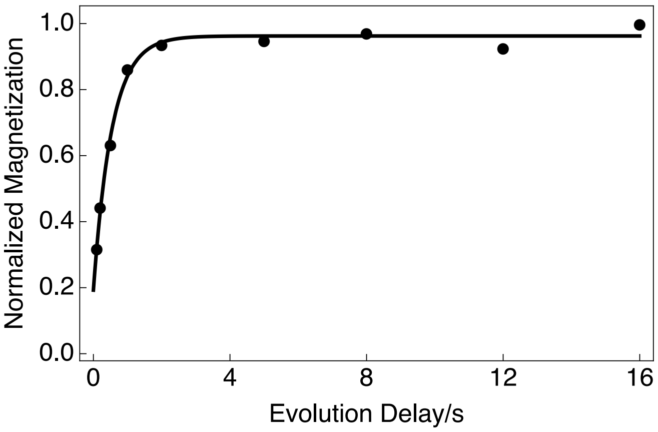

Figure **S3.3** Experimental spin-lattice relaxation curve for the ^{13}C(H)OH (methine) carbon of NH_3@**2** (7.3 mM), in $CDCl_3$ solution at 11.7 T and 25 °C, measured by saturation recovery. $T_1(^{13}C) = 0.54 \pm 0.06$ s.

4. IR spectra of CH_4@1 and NH_3@2

Experimental methods for IR spectroscopy of NH_3@**2** are described in the main paper. In addition, IR spectroscopy was performed on a sample of CH_4@**1** where the fraction of filled cages was $f = 0.65$. The remaining fraction of cages **1** were empty or contained endohedral H_2O. The thickness of the CH_4@**1** pellet was 0.21 mm. Transmission spectra using open hole as a reference were measured at temperatures of 5, 20, 40, 100, 200 and 300K from where the absorption spectra $\alpha(\omega,T)$ were calculated. No lines that could be associated with endohedral CH_4 were identified in $\alpha(\omega,T)$ and in the difference spectra $[\alpha(\omega,T) - \alpha(\omega,300K)]$. The frequencies of CH_4 vibrations in the gas phase are 1310.8 (F2), 1533.3 (E), 2916.5 (A1) and 3019.5 cm^{-1} (F2).[1]

Representative spectra at a single temperature point are given below. Figures S4.1, S4.2, and S4.3 show $\alpha(\omega,5K)$ of CH_4@**1** and NH_3@**2** between 800 and 6000 cm^{-1}, at 5 K. The IR spectrum of NH_3@**2** between 600 and 1100 cm^{-1} is given in Figure S4.4.

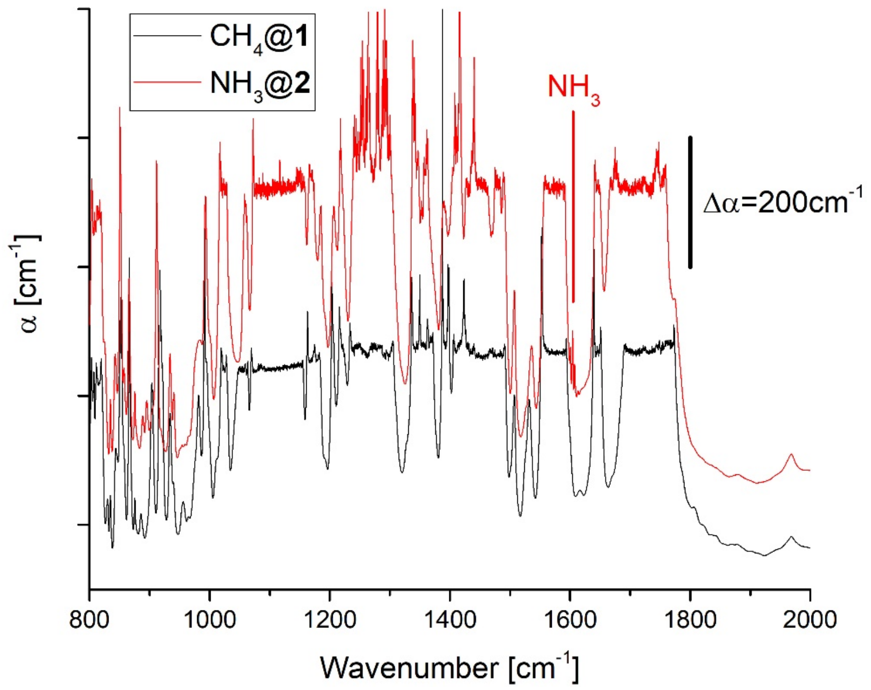

Figure S4.1 IR absorption of of CH_4@**1** and NH_3@**2** from 800 to 2000 cm^{-1} at 5 K. The spectrum of NH_3@**2** is off-set in the vertical direction. The group of endohedral NH_3 absorption lines and the vertical scale of absorption are shown by sticks. Below 1800 cm^{-1} the spectrum is dominated by strong absorption characterised by noisy and flat wavenumber dependence of α.

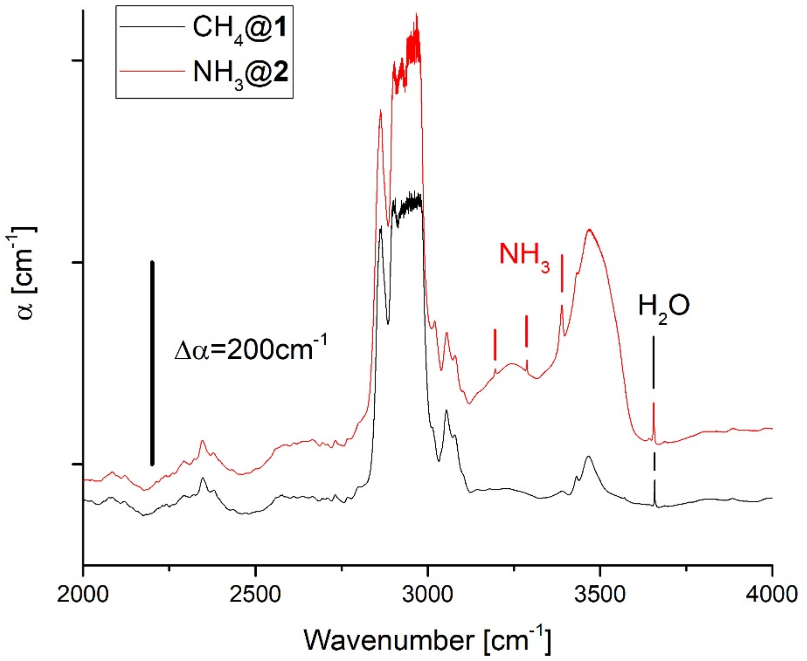

Figure S4.2 IR absorption of of CH_4@**1** and NH_3@**2** from 2000 to 4000 cm^{-1} at 5 K. The spectrum of NH_3@**2** is off-set in the vertical direction. The positions of endohedral NH_3 and H_2O absorption lines and the vertical scale of absorption are shown by sticks.

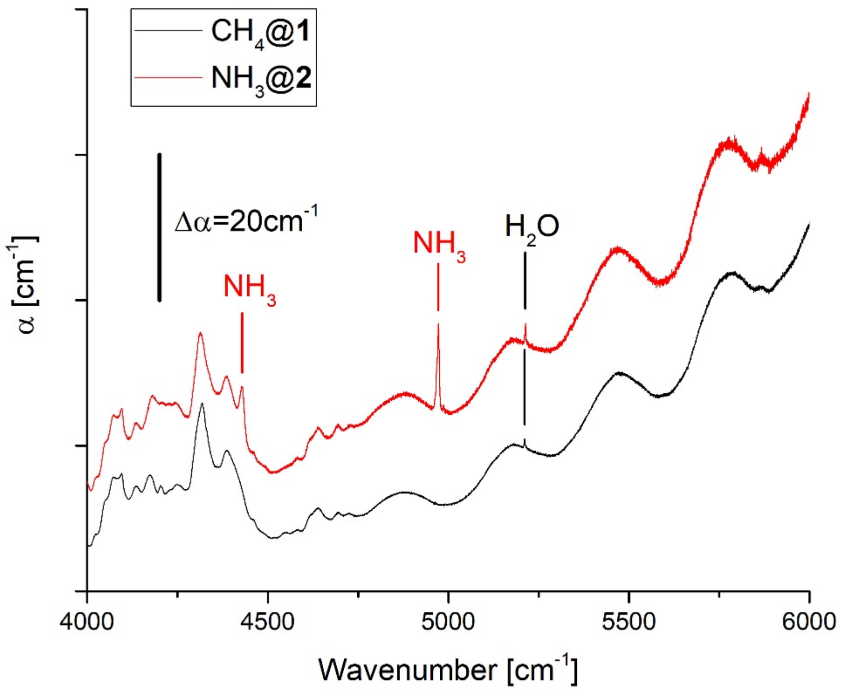

Figure S4.3 IR absorption of of CH_4@**1** and NH_3@**2** from 4000 to 6000 cm^{-1} at 5 K. The spectrum of NH_3@**2** is off-set in the vertical direction. The positions of endohedral NH_3 and H_2O absorption lines and the vertical scale of absorption are shown by sticks.

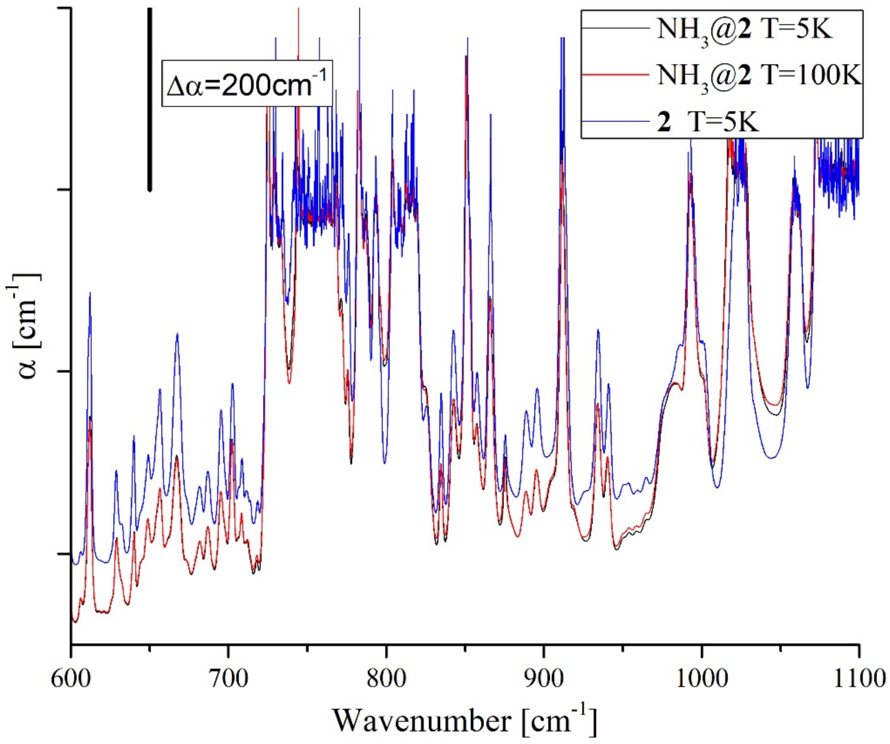

Figure S4.4 NH_3@**2** absorption at 5 K (black) and 100 K (red), and of empty **2** without NH_3 at 5 K (blue) in the spectral range around NH_3 mode ν_2 (in the gas phase at 950 cm^{-1}). Sample **2** pellet was 0.19 mm thick, endohedral H_2O was present in f = 0.35 cages and 0.65 of cages were empty.

5. DFT calculations using Gaussian 09.[2]

A model structure (**1b**) for structure **1** was used in which the 5-tert-butylpyridyl groups were replaced by methyl groups.

Table S5.1: Summary of outcomes of DFT calculations:

	E[a] Hartree	cp[b] Hartree	H (STP)[c] Hartree	S (STP)[d] J mol^{-1} K^{-1}	H (438K)[c] Hartree	S (438K)[d] J mol^{-1} K^{-1}
**1b**	-3218.679177		0.557182	803.48	0.602902	1129.35
CH_4	-40.501442		0.048787	186.07	0.050848	200.86
CH_4@**1b**	-3259.203436	0.001383	0.608328	850.97	0.656754	1196.23
CH_4@**1b**_TS	-3259.150489	0.001310	0.607624	822.59	0.655628	1164.79
NH_3	-56.54972106		0.038369	192.08		
NH_3@**1b**	-3275.257104	0.003708	0.59757	879.84		
NH_3@**1b**_TS	-3275.230019	0.003695	0.597108	823.08		
H_2O	-76.425261		0.025373	188.39		
H_2O@**1b**	-3295.126142	0.004914	0.584708	862.38		
H_2O@**1b**_TS	-3295.117442	0.005172	0.583812	831.08		

[a] E - Electronic energy calculated using M06-2X[3] with cc-pVTZ basis set[4] at M06-2X/cc-pVDZ geometry

[b] cp -Basis Set Superposition Error correction to electronic energy calculated using the counterpoise method.[5]

[c] H - Thermal correction to the electronic energy to give the enthalpy, calculated from frequency calculations at the M06-2X/cc-pVDZ level.

[d] S - entropy calculated from frequency calculations at the M06-2X/cc-pVDZ level.

For loss of CH_4 from CH_4@**1b** at STP		
Activation enthalpy	136.97	kJ mol^{-1}
Activation free energy for loss of CH_4	145.44	kJ mol^{-1}
Activation entropy for loss of CH_4	-28.41	J mol^{-1} K^{-1}

For loss of CH_4 from CH_4@**1b** at 165 °C		
Activation enthalpy	135.87	kJ mol^{-1}
Activation free energy for loss of CH_4	149.65	kJ mol^{-1}
Activation entropy for loss of CH_4	-31.47	J mol^{-1} K^{-1}

For formation of NH_3@**1b** at STP		
Complexation free energy	-24.48	kJ mol^{-1}
Activation free energy for entry of NH_3	62.32	kJ mol^{-1}

For formation of H_2O@**1b** at STP		
Complexation free energy	0.22	kJ mol^{-1}
Activation free energy for entry of H_2O	30.72	kJ mol^{-1}

Cartesian coordinates for CH_4@**1** M06-2X/cc-pVDZ

	X	Y	Z
C	3.595958	2.439968	-0.834277
C	2.27491	2.559179	-1.513949
C	1.130191	3.268865	-1.198758
S	1.189739	4.326764	0.226742
C	-0.148383	3.490801	1.040515
C	0.098691	2.827905	2.231584
C	1.415362	2.136013	2.623447
C	0.93986	0.751927	3.142906
C	1.452431	-0.51249	2.859939
C	2.878019	-0.701896	2.417513
C	3.218088	-1.999243	1.645068
C	4.384956	-1.759144	0.731504
H	5.318411	-1.464672	1.215076
C	4.333075	-1.879133	-0.591973
H	5.231106	-1.682824	-1.179654
C	3.095877	-2.204856	-1.381063
C	2.534032	-0.973242	-2.114903
C	3.026396	0.320728	-1.979742
C	4.151773	1.018752	-1.233445
C	1.954553	-2.639821	-0.467858
C	2.004463	-2.494488	0.902035
C	0.801673	-2.625685	1.690596
C	0.543513	-1.643924	2.751405
C	0.699823	-2.955304	-1.113306
C	2.173861	1.416365	-2.360746
C	1.241996	-1.179024	-2.779725
C	1.057023	1.233319	-3.120322
C	0.629903	-0.09578	-3.428931
C	0.360252	-2.28264	-2.367708
C	-0.092057	2.068183	-2.91646
C	-0.145042	2.94901	-1.820529
C	-0.324315	-3.464626	-0.315982
C	-0.274657	-3.293084	1.117795
C	-0.448294	0.887214	3.431859
C	-0.779702	-1.501403	3.195411
C	-1.286389	-0.205994	3.549549
C	-0.955452	2.120964	2.868884
C	-1.63067	-3.131226	1.591843
C	-1.418222	3.302197	0.349919
C	-2.52954	-3.208899	0.462228
C	-1.431404	3.090625	-1.11118
C	-1.708647	-3.415479	-0.714972
C	-2.039086	-2.818669	-1.914755
C	-0.995185	-2.257205	-2.750572
C	-1.225412	1.321404	-3.380681
C	-1.580587	-1.103317	-3.408958
C	-0.779347	-0.023855	-3.697906
C	-2.917392	-0.886461	-2.896808
C	-2.450948	1.531611	-2.795344
C	-2.557241	2.439535	-1.663586
C	-3.218314	-1.970061	-1.988421
C	-3.346606	0.408484	-2.60044
C	-3.98981	-1.73947	-0.854736
C	-4.101809	0.658825	-1.39705
C	-3.631812	1.91737	-0.841712
C	-3.647173	-2.383025	0.402208
C	-3.594297	2.085584	0.524863
C	-2.481312	2.788133	1.112673
C	-2.253503	2.197143	2.410818
C	-2.676265	-0.114458	3.142791
C	-3.146002	1.064907	2.572194
C	-3.050912	-1.390945	2.577042
C	-3.909598	-1.446769	1.479521
C	-4.003151	1.010391	1.412456
C	-4.396403	-0.215585	0.886228
C	-4.444415	-0.393398	-0.55362
O	5.290875	0.707076	-1.038559
O	4.188778	3.216601	-0.143961
O	3.741248	0.084675	2.71361
O	2.52466	2.535142	2.438533
C	-1.881643	-2.249721	2.625733
C	-0.692786	-0.06641	-0.027206
H	-0.775891	0.796401	0.645516
H	0.357405	-0.393188	-0.077785
H	-1.318829	-0.8864	0.352617
H	-1.035765	0.218184	-1.031242
C	3.635371	-3.027764	2.727963
H	3.880386	-3.984477	2.245991
H	4.521885	-2.654068	3.259413
H	2.831156	-3.195223	3.458973
C	3.445423	-3.312619	-2.39759
H	4.254322	-2.966166	-3.056773
H	3.790314	-4.205694	-1.857688
H	2.583074	-3.587776	-3.018966

Cartesian coordinates for CH_4@**1b** Transition state for CH_4 entry. M06-2X/cc-pVDZ
Frequency of single imaginary vibration: -46.25

	X	Y	Z
C	-3.9116	-2.4496	0.8473
C	-2.5217	-2.9408	0.6263
C	-1.4081	-3.0449	1.4537
S	-1.5443	-2.4878	3.1506
C	-0.1184	-1.4043	3.1477
C	-0.217	-0.064	3.5148
C	-1.3949	0.9451	3.4665
C	-0.7704	2.1607	2.7078
C	-1.1845	2.8302	1.5547
C	-2.6143	2.9268	1.076
C	-2.8813	2.8497	-0.4602
C	-4.0718	1.9903	-0.8012
H	-4.9972	2.2355	-0.2737
C	-4.0715	1.0277	-1.7263
H	-5.0054	0.5072	-1.9432
C	-2.8748	0.5787	-2.5344
C	-2.453	-0.8961	-2.2666
C	-3.1004	-1.7708	-1.3934
C	-4.33	-1.7367	-0.504
C	-1.6557	1.4489	-2.2059
C	-1.6577	2.4263	-1.2279
C	-0.4235	3.0601	-0.815
C	-0.1798	3.2958	0.6149
C	-0.4165	1.1063	-2.8689
C	-2.318	-2.8228	-0.7862
C	-1.1379	-1.2863	-2.7977
C	-1.1418	-3.2308	-1.3291
C	-0.6121	-2.5472	-2.4667
C	-0.1636	-0.2811	-3.2489
C	-0.0588	-3.6315	-0.4827
C	-0.0969	-3.3293	0.8928
C	0.6619	1.9793	-2.7246
C	0.6653	2.9616	-1.6692
C	0.6237	2.0714	2.9786
C	1.1549	3.4043	1.0364
C	1.5695	2.7797	2.2606
C	0.9519	0.7507	3.4765
C	2.0295	3.1277	-1.2202
C	1.1461	-1.9247	2.6277
C	2.8791	2.2583	-1.9988
C	1.1651	-2.9139	1.5324
C	2.0252	1.5673	-2.943
C	2.28	0.2612	-3.2994
C	1.1761	-0.6616	-3.4735
C	1.1304	-3.5349	-1.2755
C	1.6718	-1.962	-3.0646
C	0.7899	-2.8574	-2.5123
C	2.9987	-1.8126	-2.5041
C	2.3302	-3.2915	-0.658
C	2.3513	-3.0168	0.769
C	3.4016	-0.4369	-2.6885
C	3.3266	-2.4751	-1.3199
C	4.1863	0.1998	-1.7327
C	4.0801	-1.7921	-0.2966
C	3.4944	-2.1548	0.9838
C	3.9359	1.5892	-1.3914
C	3.4736	-1.2378	2.0074
C	2.2978	-1.1448	2.8356
C	2.2043	0.224	3.27
C	2.9141	2.2587	2.0907
C	3.2291	1.0028	2.5995
C	3.3677	2.6307	0.7694
C	4.1714	1.7632	0.0301
C	4.0335	0.0903	1.8247
C	4.5252	0.4678	0.5779
C	4.5399	-0.497	-0.5079
O	-5.443	-1.3482	-0.7121
O	-4.6224	-2.5422	1.8061
O	-3.5162	3.1583	1.8413
O	-2.4942	0.815	3.9079
C	2.2746	3.3326	0.123
C	-2.5522	0.0591	1.1382
H	-2.6784	-0.7672	1.8331
H	-3.1753	0.8634	1.5257
H	-1.4908	0.3327	1.0903
H	-2.866	-0.1706	0.1297
C	-3.2352	4.3115	-0.8542
H	-3.4416	4.3591	-1.932
H	-4.1256	4.6267	-0.2933
H	-2.4066	4.9955	-0.6178
C	-3.2347	0.7246	-4.0318
H	-4.1165	0.1094	-4.2611
H	-3.4699	1.7752	-4.2527
H	-2.4057	0.403	-4.6775

Cartesian coordinates for NH_3@**1b** M06-2X/cc-pVDZ

	X	Y	Z
C	3.62393	2.40887	-0.86708
C	2.30268	2.52658	-1.54514
C	1.15952	3.24087	-1.23326
S	1.22409	4.30982	0.18431
C	-0.12374	3.49421	1.00164
C	0.11152	2.84972	2.20497
C	1.42183	2.16533	2.6245
C	0.93632	0.78588	3.14771
C	1.4437	-0.48205	2.87595
C	2.87003	-0.68098	2.43902
C	3.20478	-1.98522	1.6777
C	4.37521	-1.7618	0.76445
H	5.30855	-1.46523	1.24699
C	4.32713	-1.90138	-0.55738
H	5.2284	-1.71861	-1.14446
C	3.09158	-2.23294	-1.34682
C	2.5407	-1.01114	-2.10457
C	3.04317	0.28088	-1.98882
C	4.1736	0.98158	-1.25385
C	1.94272	-2.64177	-0.43148
C	1.98951	-2.47813	0.93627
C	0.78437	-2.59787	1.72262
C	0.52781	-1.60833	2.77446
C	0.68891	-2.96333	-1.07712
C	2.19629	1.37654	-2.38188
C	1.2471	-1.21853	-2.76604
C	1.0762	1.18875	-3.1349
C	0.64056	-0.14086	-3.42696
C	0.35679	-2.31037	-2.34237
C	-0.06926	2.02764	-2.93521
C	-0.11678	2.91965	-1.85039
C	-0.33927	-3.45808	-0.2765
C	-0.29269	-3.26697	1.15413
C	-0.45329	0.92905	3.42699
C	-0.79569	-1.45799	3.21536
C	-1.29844	-0.15743	3.55543
C	-0.95027	2.15662	2.84489
C	-1.64892	-3.09677	1.62294
C	-1.39237	3.30257	0.30891
C	-2.54489	-3.1858	0.49255
C	-1.40363	3.07572	-1.14783
C	-1.72217	-3.4109	-0.6796

C	-2.04648	-2.82726	-1.88671
C	-0.99775	-2.28171	-2.72606
C	-1.20542	1.28372	-3.39188
C	-1.57572	-1.13407	-3.39801
C	-0.76762	-0.06457	-3.69526
C	-2.91143	-0.90413	-2.89215
C	-2.4293	1.50716	-2.8142
C	-2.53173	2.42711	-1.69482
C	-3.22147	-1.97465	-1.97345
C	-3.3326	0.39455	-2.61045
C	-3.99603	-1.72739	-0.84543
C	-4.09045	0.6631	-1.41427
C	-3.61254	1.92349	-0.8718
C	-3.65918	-2.35689	0.42008
C	-3.58002	2.10831	0.49197
C	-2.46303	2.80743	1.07383
C	-2.24517	2.23256	2.3801
C	-2.68712	-0.06418	3.14313
C	-3.147	1.11009	2.55384
C	-3.06585	-1.34452	2.58897
C	-3.92001	-1.40789	1.48706
C	-4.0001	1.04757	1.39148
C	-4.39884	-0.18168	0.87744
C	-4.44442	-0.37625	-0.56075
O	5.31185	0.66674	-1.05796
O	4.22001	3.18829	-0.18222
O	3.7349	0.10507	2.73119
O	2.53384	2.56492	2.4592
C	-1.8994	-2.20648	2.64921
C	3.61188	-3.00773	2.77025
H	3.85048	-3.97031	2.29689
H	4.5002	-2.63628	3.30022
H	2.80501	-3.16278	3.5011
C	3.43683	-3.3627	-2.34022
H	4.25263	-3.03514	-3.00045
H	3.77053	-4.2489	-1.78229
H	2.57546	-3.64073	-2.96165
N	-0.9251	-0.07436	-0.20821
H	-0.82738	0.77035	0.36584
H	0.0187	-0.47863	-0.2266
H	-1.46435	-0.71943	0.38009

Cartesian coordinates for NH_3@**1b** Transition State for NH_3 entry/exit M06-2X/cc-pVDZ
Frequency of single imaginary vibration: -97.46

	X	Y	Z
C	-3.746933	-2.479197	-0.056418
C	-2.451927	-2.939733	-0.616118
C	-1.329017	-3.402151	0.052986
S	-1.476888	-3.589466	1.821656
C	-0.034714	-2.661086	2.276985
C	-0.161405	-1.614266	3.187873
C	-1.394553	-0.727236	3.427156
C	-0.811149	0.712368	3.336593
C	-1.260659	1.784735	2.563891
C	-2.689649	1.878944	2.10522
C	-2.985273	2.641649	0.784981
C	-4.160208	2.02599	0.078071
H	-5.085797	1.97261	0.654892
C	-4.141963	1.602366	-1.185132
H	-5.062233	1.215222	-1.622613
C	-2.93711	1.574755	-2.086221
C	-2.47829	0.130246	-2.419865
C	-3.078923	-1.02425	-1.929579
C	-4.236323	-1.323651	-0.999598
C	-1.748199	2.26861	-1.415547
C	-1.767971	2.712705	-0.102866
C	-0.543607	3.106734	0.558668
C	-0.294842	2.673362	1.943241
C	-0.50234	2.251974	-2.147081
C	-2.291353	-2.233646	-1.848513
C	-1.171382	0.023782	-3.077679
C	-1.123781	-2.359659	-2.537993
C	-0.619052	-1.251054	-3.289019
C	-0.22418	1.150557	-3.070592
C	-0.018182	-3.066048	-1.958603
C	-0.028376	-3.401589	-0.588775
C	0.560168	2.992327	-1.627873
C	0.545149	3.413096	-0.248212
C	0.582885	0.580569	3.584345
C	1.040054	2.63141	2.380914
C	1.490585	1.56121	3.225108
C	0.972389	-0.81472	3.498584
C	1.907051	3.394839	0.235955
C	1.227003	-2.863777	1.571257
C	2.773818	2.972469	-0.838271
C	1.240206	-3.273595	0.154186
C	1.931233	2.737662	-1.992704
C	2.208388	1.711341	-2.869526
C	1.122547	0.925543	-3.423317
C	1.157987	-2.611289	-2.640695
C	1.646109	-0.415412	-3.599352
C	0.787328	-1.479454	-3.467668
C	2.976306	-0.487525	-3.031544
C	2.366319	-2.630332	-1.991903
C	2.411998	-2.998345	-0.587513
C	3.349146	0.844941	-2.613539
C	3.335054	-1.584055	-2.245882
C	4.132253	1.033069	-1.479359
C	4.090047	-1.384145	-1.033077
C	3.53679	-2.27789	-0.028946
C	3.852005	2.135241	-0.574871
C	3.516149	-1.891665	1.290841
C	2.358425	-2.204349	2.089351
C	2.234806	-1.156351	3.071483
C	2.852012	1.213639	2.857147
C	3.217424	-0.127444	2.784585
C	3.277267	2.128609	1.822477
C	4.097739	1.688205	0.783896
C	4.033976	-0.594423	1.691006
C	4.493476	0.294648	0.723371
C	4.517327	-0.110989	-0.671215
O	-5.363088	-0.915465	-0.972618
O	-4.349613	-2.850253	0.911115
O	-3.591228	1.426351	2.765801
O	-2.530948	-1.079325	3.535401
C	2.154726	3.002591	1.535327
C	-3.379443	4.073285	1.23746
H	-3.613562	4.682519	0.353839
H	-4.265884	4.016608	1.884364
H	-2.563342	4.555153	1.795676
C	-3.297949	2.304675	-3.400593
H	-4.155761	1.80668	-3.874575
H	-3.570016	3.346733	-3.181226
H	-2.456735	2.29929	-4.107311
N	-1.776369	-0.458694	0.526804
H	-0.892339	-0.713151	0.966406
H	-2.471784	-0.403424	1.272683
H	-1.643067	0.480709	0.162323

Cartesian coordinates for H_2O@**1b** M06-2X/cc-pVDZ

	X	Y	Z
C	3.66794	2.42077	-0.79813
C	2.34849	2.57274	-1.47208
C	1.20813	3.27283	-1.12041
S	1.27836	4.27046	0.34669
C	-0.0817	3.43686	1.12702
C	0.13665	2.75401	2.3108
C	1.43508	2.04457	2.72069
C	0.93104	0.65518	3.19534
C	1.43053	-0.6062	2.88211
C	2.85971	-0.80312	2.45545
C	3.1863	-2.06597	1.62253
C	4.35885	-1.80161	0.7226
H	5.2907	-1.5275	1.22099
C	4.31709	-1.88873	-0.60425
H	5.22192	-1.68624	-1.17894
C	3.08569	-2.18777	-1.4131
C	2.55212	-0.94092	-2.14265
C	3.07047	0.34089	-1.99577
C	4.20763	1.0061	-1.23769
C	1.92855	-2.61787	-0.51809
C	1.96986	-2.51396	0.85533
C	0.75944	-2.66077	1.63098
C	0.50368	-1.71752	2.72547
C	0.67596	-2.90535	-1.17998
C	2.23545	1.45808	-2.35536
C	1.2578	-1.11467	-2.81421
C	1.1122	1.30478	-3.11222
C	0.66521	-0.01205	-3.44839
C	0.35554	-2.2095	-2.42427
C	-0.02835	2.14398	-2.87741
C	-0.07049	2.9852	-1.75062
C	-0.36038	-3.42478	-0.40719
C	-0.32134	-3.29361	1.02928
C	-0.46006	0.80163	3.46314
C	-0.82433	-1.5695	3.15531
C	-1.31801	-0.27911	3.54232
C	-0.93843	2.05138	2.91634
C	-1.67882	-3.13318	1.49518
C	-1.34692	3.28304	0.42366
C	-2.56764	-3.17369	0.35899
C	-1.3553	3.1163	-1.03893
C	-1.74018	-3.35726	-0.81512
C	-2.05573	-2.73355	-2.00348
C	-0.99762	-2.16396	-2.81589
C	-1.17134	1.42517	-3.36023
C	-1.56374	-0.98929	-3.45216
C	-0.74395	0.08435	-3.71311
C	-2.9019	-0.76955	-2.94302
C	-2.39513	1.63268	-2.77009
C	-2.49185	2.50643	-1.6139
C	-3.22571	-1.87165	-2.06532
C	-3.31375	0.52298	-2.61379
C	-3.99874	-1.65637	-0.92961
C	-4.07494	0.75401	-1.41013
C	-3.58564	1.98741	-0.81765
C	-3.6729	-2.33381	0.3126
C	-3.55352	2.11638	0.55148
C	-2.42873	2.77574	1.16169
C	-2.22824	2.15589	2.44676
C	-2.70217	-0.15764	3.12333
C	-3.145	1.04095	2.57537
C	-3.08837	-1.41092	2.51782
C	-3.93597	-1.42606	1.41184
C	-3.9925	1.02995	1.40863
C	-4.40086	-0.17433	0.84791
C	-4.43902	-0.31376	-0.59646
O	5.34384	0.67546	-1.05804
O	4.26692	3.1694	-0.08232
O	3.73243	-0.04985	2.80526
O	2.554	2.4341	2.57602
O	-1.3713	-0.19641	0.16492
H	-0.49839	-0.46328	0.49561
H	-1.21042	-0.05518	-0.78221
C	-1.93021	-2.28296	2.55182
C	3.43047	-3.29078	-2.43683
H	4.25352	-2.95109	-3.08182
H	3.75323	-4.19518	-1.9022
H	2.57136	-3.54467	-3.07186
C	3.58684	-3.14968	2.65732
H	3.82196	-4.08543	2.13143
H	4.4752	-2.8127	3.20973
H	2.77674	-3.34074	3.3762

Cartesian coordinates for H_2O@**1b** Transition State for entry / exit of H_2O
Frequency of single imaginary vibration: -53.90

	X	Y	Z
C	3.729504	2.410399	-0.5616
C	2.419615	2.692246	-1.207505
C	1.285126	3.320853	-0.717537
S	1.391383	4.012912	0.918931
C	-0.032345	3.173286	1.568315
C	0.114911	2.362888	2.687159
C	1.369653	1.558779	3.053831
C	0.810689	0.151792	3.37304
C	1.278271	-1.067485	2.887198
C	2.704665	-1.221804	2.440495
C	3.022241	-2.316821	1.398236
C	4.206673	-1.915494	0.567748
H	5.128558	-1.712352	1.116242
C	4.193767	-1.83418	-0.760481
H	5.112806	-1.570834	-1.284997
C	2.985252	-2.030729	-1.631546
C	2.508113	-0.707204	-2.263671
C	3.088973	0.533018	-2.026709
C	4.242957	1.067052	-1.204762
C	1.801009	-2.547167	-0.818437
C	1.815153	-2.632451	0.558196
C	0.592737	-2.86444	1.290527
C	0.330058	-2.101031	2.518609
C	0.559847	-2.722003	-1.531901
C	2.280928	1.709814	-2.234077
C	1.210818	-0.768829	-2.945939
C	1.136248	1.669003	-2.970872
C	0.652861	0.412997	-3.456254
C	0.27558	-1.874936	-2.689271
C	0.01673	2.487688	-2.608317
C	-0.001653	3.158407	-1.370503
C	-0.50129	-3.319025	-0.851868
C	-0.488034	-3.383215	0.587783
C	-0.584745	0.316029	3.599102
C	-1.005448	-1.975288	2.937531
C	-1.475317	-0.737444	3.493865
C	-1.002759	1.640354	3.184015
C	-1.851858	-3.259695	1.049615
C	-1.2895	3.176555	0.82989
C	-2.718015	-3.12358	-0.098206
C	-1.285277	3.218114	-0.644291
C	-1.871246	-3.169898	-1.272104
C	-2.152899	-2.392903	-2.375443
C	-1.068884	-1.75166	-3.09397
C	-1.14437	1.87189	-3.182095
C	-1.599615	-0.495484	-3.587254
C	-0.751128	0.578952	-3.707476
C	-2.937305	-0.304451	-3.066264
C	-2.364068	2.034877	-2.574125
C	-2.438719	2.741712	-1.306975
C	-3.301582	-1.500772	-2.340993
C	-3.318227	0.94523	-2.575138
C	-4.092834	-1.416124	-1.199523
C	-4.087167	1.038057	-1.357325
C	-3.56033	2.16053	-0.597787
C	-3.806218	-2.260023	-0.051964
C	-3.550893	2.108994	0.777461
C	-2.409681	2.630953	1.484813
C	-2.270691	1.85012	2.690197
C	-2.841285	-0.512044	3.051628
C	-3.232284	0.764318	2.656777
C	-3.2464	-1.658924	2.271027
C	-4.067705	-1.498293	1.155225
C	-4.048149	0.937349	1.478885
C	-4.483515	-0.168201	0.754519
C	-4.499655	-0.114985	-0.69755
O	5.377502	0.701553	-1.08408
O	4.324157	3.016342	0.284701
O	3.588853	-0.566142	2.932683
O	2.50447	1.919082	2.935299
O	1.293704	0.33755	0.328188
H	1.980405	0.881736	0.74513
H	0.486269	0.843414	0.501942
C	-2.111831	-2.562294	2.211979
C	3.351489	-3.031705	-2.750635
H	4.202128	-2.643525	-3.329349
H	3.640443	-3.992861	-2.302398
H	2.511154	-3.201336	-3.437528
C	3.413193	-3.562672	2.238634
H	3.655597	-4.395298	1.564069
H	4.293657	-3.324608	2.851714
H	2.592542	-3.872023	2.902385

5. References

[1] J. M. G. Niederer, The Infrared Spectrum of Methane, *Diss. ETH Nr.*, **2011**, 19829.

[2] Gaussian 09, Revision D.01, M. J. Frisch, G. W. Trucks, H. B. Schlegel, G. E. Scuseria, M. A. Robb, J. R. Cheeseman, G. Scalmani, V. Barone, B. Mennucci, G. A. Petersson, H. Nakatsuji, M. Caricato, X. Li, H. P. Hratchian, A. F. Izmaylov, J. Bloino, G. Zheng, J. L. Sonnenberg, M. Hada, M. Ehara, K. Toyota, R. Fukuda, J. Hasegawa, M. Ishida, T. Nakajima, Y. Honda, O. Kitao, H. Nakai, T. Vreven, J. A. Montgomery, Jr., J. E. Peralta, F. Ogliaro, M. Bearpark, J. J. Heyd, E. Brothers, K. N. Kudin, V. N. Staroverov, T. Keith, R. Kobayashi, J. Normand, K. Raghavachari, A. Rendell, J. C. Burant, S. S. Iyengar, J. Tomasi, M. Cossi, N. Rega, J. M. Millam, M. Klene, J. E. Knox, J. B. Cross, V. Bakken, C. Adamo, J. Jaramillo, R. Gomperts, R. E. Stratmann, O. Yazyev, A. J. Austin, R. Cammi, C. Pomelli, J. W. Ochterski, R. L. Martin, K. Morokuma, V. G. Zakrzewski, G. A. Voth, P. Salvador, J. J. Dannenberg, S. Dapprich, A. D. Daniels, O. Farkas, J. B. Foresman, J. V. Ortiz, J. Cioslowski, and D. J. Fox, Gaussian, Inc., Wallingford CT, **2013**.

[3] Y. Zhao and D. G. Truhlar, *Theor. Chem. Acc.*, **2008**, *120*, 215 - 241.

[4] T. H. Dunning Jr., *J. Chem. Phys.*, **1989**, *90*, 1007 - 1023.

[5] S. F. Boys and F. Bernardi, *Mol. Phys.*, **1970**, *19*, 553 - 566.
